# Breeding for adaptation to climate change: genomic selection for drought response in a white spruce multi‐site polycross test

**DOI:** 10.1111/eva.13348

**Published:** 2022-02-28

**Authors:** Jean‐Philippe Laverdière, Patrick Lenz, Simon Nadeau, Claire Depardieu, Nathalie Isabel, Martin Perron, Jean Beaulieu, Jean Bousquet

**Affiliations:** ^1^ 4440 Canada Research Chair in Forest Genomics Institute for Systems and Integrative Biology and Centre for Forest Research Université Laval Québec QC Canada; ^2^ Natural Resources Canada Canadian Forest Service Canadian Wood Fibre Centre Québec QC Canada; ^3^ Natural Resources Canada Canadian Forest Service Laurentian Forestry Centre Québec QC Canada; ^4^ Direction de la Recherche Forestière Ministère des Forêts, de la Faune et des Parc du Québec Québec QC Canada

**Keywords:** adaptation, conifer, dendrochronology, drought resistance, multi‐trait selection, tree rings

## Abstract

With climate change, increasingly intense and frequent drought episodes will be affecting water availability for boreal tree species, prompting tree breeders and forest managers to consider adaptation to drought stress as a priority in their reforestation efforts. We used a 19‐year‐old polycross progeny test of the model conifer white spruce (*Picea glauca*) replicated on two sites affected by distinct drought episodes at different ages to estimate the genetic control and the potential for improvement of drought response in addition to conventional cumulative growth and wood quality traits. Drought response components were measured from dendrochronological signatures matching drought episodes in wood ring increment cores. We found that trees with more vigorous growth during their lifespan resisted better during the current year of a drought episode when the drought had more severe effects. Phenotypic data were also analyzed using genomic prediction (GBLUP) relying on the genomic relationship matrix of multi‐locus gene SNP marker information, and conventional analysis (ABLUP) based on validated pedigree information. The accuracy of predicted breeding values for drought response components was marginally lower than that for conventional traits and comparable between GBLUP and ABLUP. Genetic correlations were generally low and nonsignificant between drought response components and conventional traits, except for resistance which was positively correlated to tree height. Heritability estimates for the components of drought response were slightly lower than for conventional traits, but similar single‐trait genetic gains could be obtained. Multi‐trait genomic selection simulations indicated that it was possible to improve simultaneously for all traits on both sites while sacrificing little on gain in tree height. In a context of rapid climate change, our results suggest that with careful phenotypic assessment, drought response may be considered in multi‐trait improvement of white spruce, with accelerated screening of large numbers of candidates and selection at young age with genomic selection.

## 1 INTRODUCTION

Spanning the surface of the Earth for the last 300 million years (Gernandt et al., 2011), conifers had to cope and adapt to diverse local climatic conditions. Extreme climatic events, such as drought episodes, have been an important driver in the shaping of selection and dispersion of adapted genotypes to these conditions as water availability has proven to be a key determinant of survival and reproduction (Allen et al., 2010; Williams, 2009). Thus, conifers have developed multiple functional traits to cope with periods of low water availability (Aubin et al., 2016). However, the temperate–boreal forest is being and will be even more impacted by the increasing effects of climate change. Some of the common projected future changes are the rises of the carbon dioxide (CO_2_) concentration, mean annual temperature, and annual precipitation for some regions, which could mistakenly lead to projected increased forests productivity when only considering mean annual climatic parameters (D'Orangeville et al., 2018; Price et al., 2013). Indeed, precipitation episodes are projected to be constrained to shorter periods with more short‐term instability in climate, leading to increasing intensity and frequency of extreme climatic events, such as droughts episodes and heat waves (IPCC, 2021). Extreme climatic events are therefore projected to be an important driver of the temperate–boreal forest growth compared to average conditions (Frelich et al., 2021; Germain & Lutz, 2020). These fluctuations and more adverse climatic conditions will affect the adaptive capacity and productivity of many tree species (Price et al., 2013). The consequences will be a direct outcome of climate or a consequence from increased competition from more adapted or opportunistic species (Zhang et al., 2015). Forest growth reductions are projected alongside tree mortality and die‐off, even in environments that are currently not water‐limited (Allen et al., 2010). In comparison with annual plants which might present a faster adaptive response at the genetic level (Dickman et al., 2019), the long generation time of conifers, such as for white spruce (*Picea glauca* [Moench] Voss) (Bouillé & Bousquet, 2005), makes them locally vulnerable to rapid environmental changes including the predicted increasing frequency and intensity of drought episodes.

White spruce is a cornerstone tree species of the temperate–boreal forest, with transcontinental natural distribution ranging from Newfoundland and Labrador to Alaska in North America (Burns & Honkala, 1990). It is intensively reforested and highly important to the Canadian wood industry for pulp and lumber production, being valued for its superior wood mechanical properties (Middleton & Zhang, 2009). By the end of the century, white spruce is projected to be highly unsuited on 20% of its current habitat, with 70% of it becoming under less suitable climatic conditions in Quebec (Périé et al., 2014). In addition, because of climate warming during the last half‐century, it has been shown that local white spruce populations are already misadapted genetically to their local conditions (Andalo et al., 2005), which prompted the need for seed source transfer modelling to better guide reforestation efforts (Rainville & Beaulieu, 2005). For drought stress, dendrochronological studies in white spruce have underlined the negative impact of dry conditions experienced during the current and previous seasons on radial growth in the following growing seasons (Barber et al., 2000; Chen et al., 2017; Depardieu et al., 2020; Sang et al., 2019). Such impacts have also been observed for many other tree species (Girardin et al., 2016; Gazol et al., 2017; Hogg et al., 2017; Housset et al., 2018). A long‐term common garden study recently highlighted the existence of local genetic adaptation of drought stress response in white spruce from the wide variation observed among the several dozen tested seed sources originating from dryer to more humid climatic conditions (Depardieu et al., 2020). These observations prompted the need to look further into the genetic variation of drought response in this species and how it could be considered in tree breeding and reforestation efforts.

To avoid stress under intense drought episodes, reforested tree species could benefit from breeding programs aiming to improve drought response for future planted stock. White spruce is the subject of intensive genetic improvement programs in several jurisdictions of Canada (Beaulieu, 1996; Mullin et al., 2011). Traits that have been the traditional focus for genetic improvement of this species and other conifers are survival rate after plantation, height, and diameter growth (Mullin et al., 2011). Phenology as well as wood mechanical proprieties and quality have been considered as well in white spruce (Beaulieu, 1996; Beaulieu et al., 2014; Li et al., 1993). However, several Canadian spruce breeding programs are currently aiming to expand their breeding objectives to better integrate resilience to biotic and abiotic factors. For example, accuracy of predictions for breeding values has been recently studied while selecting for weevil resistance, growth, and wood quality for the introduced Norway spruce in eastern Canada (*Picea abies* (L.) Karst.) (Lenz, Nadeau, Mottet, et al., 2020), and for spruce budworm resistance, growth, and wood quality traits in white spruce (Beaulieu et al., 2020). In the face of climate change, the use of an augmented number of breeding objectives in conifer breeding programs reflects a desire to restock forests with fast growing and high‐quality material which is also well adapted to future climatic conditions. Such multiple objectives require efficient and accurate determination of the genetic merit of large cohorts of candidate trees.

Genomic selection (GS) is one promising strategy that could help integrate rapidly multiple breeding objectives into spruce breeding programs (Park et al., 2016), including drought response. Multi‐trait GS has been shown to represent an accurate predictive tool (Lenz, Nadeau, Mottet, et al., 2020) with increased economic benefits (Chamberland et al., 2020) compared with more conventional pedigree‐based selection in white spruce breeding programs, by reducing much the time needed to complete a breeding cycle. GS uses genetic values based on dense marker information covering the genome (Meuwissen et al., 2001), which can be conducted at a very early age on large cohorts without the need to phenotype candidate trees (Bousquet et al., 2021; Park et al., 2016). Given this and the importance of considering multi‐trait selection strategies, there is an opportunity to use genomic information from genomic profiles to build GS models and estimate more precisely the genetic control of drought response and better understand the genetic correlations with more conventional traits. Considering the evidence of diminished growth and poorer survival rate of less resilient trees after drought episodes (DeSoto et al., 2020) and with the projected increased frequency and intensity of these, tree breeders are becoming under pressure to consider drought response into their selection and breeding scenarios.

The main objective of this study was to evaluate the efficiency of genomic prediction for drought response in an advanced‐breeding population of white spruce established on two sites and derived from a polycross mating design. The specific objectives were to: (1) quantify the drought response of trees in a genetic trial repeated on two contrasting environments, (2) assess the genetic control of drought response traits and their relationships with more conventional traits (diameter at breast height, tree height, wood density, and acoustic velocity as a proxy for wood stiffness), and (3) understand the implications of conventional and genomic selection on the expected genetic gains obtained for drought response in multi‐trait selection schemes. By doing so, we aimed at providing data to tree breeders to inform them on the potential for enhancing drought response in the model conifer white spruce and the consequences of improving these traits on more conventional traits.

## 2 MATERIAL AND METHODS

### 2.1 Study area

This study was conducted in a polycross genetic trial replicated on two contrasting environments in the province of Québec, Canada: in Normandin (N 48°50’, W 72°30’, elevation: 122 m), and Watford (N 46°13’, W 70°31’, elevation: 300m), respectively, located in the more northerly balsam fir‐yellow birch domain and in the more southerly warmer sugar maple‐yellow birch bioclimatic domain characterized by more favorable growth conditions. The field trial was established in 1997 using 2‐year‐old seedlings raised in greenhouse at the Laurentian Forestry Centre (Quebec City, Canada) from February to June 1995 and then moved to the nursery of the Valcartier Forest Experiment Station near Quebec City. A total of 38 families obtained from a polycross mating scheme were represented. The polymix contained an equal volume of pollen from 19 fathers. The experimental design consisted of a randomized complete block with four blocks. Four trees per block and family were planted in noncontiguous single tree plots following an interlocked design to allow for systematic thinning. The initial spacing between trees was 2.0m x 2.0m. Wood cores were extracted from trees in 2015, at age 18 since plantation, from the south‐facing side of the trees and then stored in a freezer, conditioned to 7% moisture, and then cut to a 1.68 mm thickness for X‐ray analyses (see below). Diameter at breast height (DBH), tree height, and acoustic velocity were measured in 2016 at age 19 since plantation. Acoustic velocity is an indirect assessment trait for wood stiffness in standing trees and was measured using the Hitmam ST300 tool (Fibre‐gen, New Zealand) (Lenz et al., 2013). In the rest of this report, together these traits will be referred to as conventional cumulative traits since they represent trait attributes over the whole trees’ lifespan, contrary to components of drought response which estimation is restricted to the years around a drought episode (see below). A total of 281 trees were phenotyped for the Normandin study site and 279 for the Watford site, representing from six to nine trees per family at each site.

### 2.2 Tree‐ring data

Ring width and wood density were measured from wood cores using a Quintek X‐Ray measurement system (TN, USA) (Lenz et al., 2017). Annual basal area increment (BAI) values were inferred from ring‐width values. Dating validation of cores’ ring‐width chronologies was conducted using COFECHA (Holmes, 1983). Every core chronology whose correlation with its own site was below 0.2 was visually checked and then shifted to a matching dating. Nine cores were removed after failing to precisely adjust the chronology. Individual detrending of ring series was performed on BAI values with a 0.7 frequency response (f) spline curve using dplR package (Bunn, 2010) under R (R Core Team, 2019). Yearly growth indices (the ratios of raw values over the values of the detrending curves) were then extracted from the spline curve. For detrending, the dataset was subsetted to years 2003–2015 to ensure that at least 50% of individuals per site were used. Raw and detrended mean values for both sites are provided in Table S1.

### 2.3 Climatic data

To detect possible drought stress conditions and relationships with tree growth, local climatic data were generated using BioSIM 11 (Régnière et al., 2017). The daily and monthly mean climatic variables from 1995 to 2015 regarding temperature, precipitation, relative humidity, and vapor pressure deficits were simulated as described in Depardieu et al. (2020). Monthly Drought Code (DC) and Soil Moisture Index (SMI) were then simulated using the Forest Weather Index (FWI) Drought Code and Soil Moisture Index models of BioSIM. The average monthly value of each drought index was centered and scaled (subtracted from the monthly average and divided by the standard deviation), for the period 1985–2015. Centered and scaled values were then graphically compared to mean growth chronologies to identify drought years and visualize the impact of drought episodes on growth. Since DC and SMI values were highly correlated (*r* = 0.81, *p* < 0.001 at Normandin and *r* = 0.82, *p* < 0.001 at Watford), and given that both indices led to similar conclusions, we only show data and results regarding DC to detect years with drought stress signals at each site. Raw monthly DC values used for scaling are presented in Figure S1. Monthly SMI, total precipitation, and mean temperature values are presented in Figure S6.

### 2.4 Correlations between radial growth and monthly drought code values

To assess the relationship between radial growth and water availability across years 2005–2015, we estimated the correlation between detrended BAI and monthly DC values for both current and preceding year. For each site, family robust mean chronologies were generated using the "chron" function of the dplR package. The correlations between the mean chronologies and DC values then were calculated using a bootstrap approach with the "dcc" function of the treeclim package (Zang & Biondi, 2015).

### 2.5 Components of drought response

To improve the estimation of tree response to drought episodes, Lloret et al. (2011) proposed four indices that quantify growth responses at specific periods. These indices quantify growth loss due to a drought episode, growth increase after the episode as well as the general capacity of trees to reach predisturbance growth level. The proposed indices termed resistance, recovery, resilience, and relative resilience were used as drought response traits in this study on white spruce. The components of tree resilience to drought at the individual‐tree level were generated using the pointRes R package (van der Maaten‐Theunissen et al., 2015) to quantify the growth response to drought stress. To avoid any confusion, we will refer herein to these four components using the expression “components of drought response” instead of “components of drought resilience”. The resistance component of drought response was calculated as the ratio of the BAI observed during the drought episode to the predrought mean BAI. It represents the growth loss associated with the period of drought. The recovery component was calculated as the ratio of the postdrought mean BAI to the BAI observed during the drought. It represents the growth increase after the drought stress. The resilience component was calculated as the ratio of the postdrought mean BAI to the predrought mean BAI. It represents the capacity to reach predrought performance after the disturbance. Finally, the relative resilience component was obtained by subtracting the resistance component from the resilience component of drought response. It represents the resilience component weighted by the growth loss due to the drought episode. Since drought episodes are known to affect tree growth over multiple years, the postdrought period was set to 3 years and the reference predrought period to 2 years at both study sites. A summary of all traits included in this study is presented in Table 1. Given that the Watford plantation test experienced a thinning in the fall of 2012, the same year that a drought episode was identified during the summer growing season (see Results), the estimation of the postdrought components of drought response could not be estimated given the plantation thinning effects on subsequent growth. Thus, only the resistance component of drought response could be evaluated for the drought episode experienced at this site.

**TABLE 1:** Description, means, standard deviations (SD), and phenotypic coefficient of variations (CV_P_) of white spruce traits assessed at both Normandin and Watford study sites

Types of trait	Traits		Normandin site				Watford site^d^			
		Units	Age after plantation	Mean	SD	CV_p_(%)	Age after plantation	Mean	SD	CV_p_(%)
Drought response	Recovery^a^	‐	13–16	1.1	0.2	21.1	‐	‐	‐	‐
Drought response	Relative resilience^a^	‐	11–16	0.1	0.3	218.2	‐	‐	‐	‐
Drought response	Resilience^a^	‐	11–16	1.3	0.4	28.2	‐	‐	‐	‐
Drought response	Resistance^a^	‐	11–13	1.1	0.2	18.4	13–15	0.9	0.2	21.9
Growth	Height	cm	19	784.0	158.4	20.2	19	1099.8	120.1	10.9
Growth	DBH^b^	mm	19	126.3	24.7	19.6	19	160.0	21.4	13.4
Wood quality	Acoustic velocity	km/s	19	3.0	0.4	13.3	19	3.2	0.3	10.7
Wood quality	Wood density	kg/m^3^	18	382.6	28.9	7.5	18	368.9	28.8	7.8
Wood quality	EW density^b^	kg/m^3^	18	342.3	25.8	7.5	18	326.9	23.8	7.3
Wood quality	LW density^b^	kg/m^3^	18	636.0	43.1	6.8	18	651.4	44.1	6.8
Growth	EW area^b^, ^c^	mm^2^	18	9185.7	3869.9	42.1	18	14683.9	4294.8	29.2
Growth	LW area^b^, ^c^	mm^2^	18	1518.1	586.3	38.6	18	2266.2	592.5	26.1

^a^ See section “Components of drought response” in Material and Methods for the full description and estimation of drought response traits.

^b^ Abbreviations: DBH, diameter at breast height; EW, earlywood; LW, latewood.

^c^ Area traits refer to the annual basal area increment (BAI).

^d^ Given that the Watford plantation test experienced a thinning in the fall of 2012, the same year that a drought episode was identified during the summer growing season (see Results), the postdrought components of drought response could not be estimated given the plantation thinning effects on subsequent growth. Thus, only the resistance component of drought response could be evaluated for the drought episode experienced at this site.

### 2.6 Genotyping assay and paternity recovery

Genomic profiles were obtained for each tree from genotyping with an Infinium iSelect SNP array (Illumina, California) described in Lenz, Nadeau, Azaiez, et al. (2020). After quality filters, data from 4091 high‐quality Mendelian single‐nucleotide polymorphisms (SNPs) with little or no missing data and representing as many distinct gene loci well dispersed over the white spruce genome (Lenz, Nadeau, Azaiez, et al., 2020) were used for GS analyses. Pedigree informed by paternity recovery was also obtained from Lenz, Nadeau, Azaiez, et al. (2020). Paternal assignment and pedigree verification were conducted so that an informed and corrected pedigree information was also available for conventional ABLUP analyses. In doing so, a total of 347 genetically distinct full‐sib families implicating 38 mothers and 19 fathers could be recovered, in agreement with the 38 families obtained by polycross mating and the 19 fathers used in the pollen polymix to sire the female trees.

### 2.7 Statistical model

An individual‐tree linear mixed model (known as the “*animal model”) was fitted using ASRem*l‐R v.4.1 (Butler et al., 2017) using the conventional pedigree‐based relationships matrix (***A***, ABLUP method) and the realized additive genomic relationship matrix (***G***, GBLUP method). The inverse of the ***A*** matrix was computed using the "Ainverse" function from the ASReml‐R package. The ***G*** matrix was generated using the "A.mat" function from the rrBLUP package (Endelman and Jannink, 2012), which is equivalent to the equation described by VanRaden (2008). Because the two study sites suffered drought episodes that differed according to the year of occurrence, severity of effects on BAI, developmental stage of the trees, and the test plantations, the possibility of different trees’ drought response between sites was considered (see Climate–growth relationships in Results), Thus, a cautious approach was adopted where each site was analyzed separately, thus an individual‐tree linear mixed model was fitted per site and per trait such as:

(1) y=Xβ+Za+e

where ***y*** is the phenotype, **β** is a vector of fixed effects, including the overall mean and the block effect, ***a*** is the random additive genetic effect, where ***a*** ~ *N* (**0**, σ^2^_a_ ***A***), and **e** is the random residual error term, where ***e*** ~ *N* (**0**, σ^2^_e_ ***I***_**e**_). **A** is the pedigree‐based relationship matrix (ABLUP), which is replaced by the realized additive genomic relationship matrix (***G***) for the GBLUP method. The ***X*** and ***Z*** matrices are incidence matrices of their corresponding effects and the ***I***_**e**_ matrix is an identity matrix of its proper dimensions. For each trait, narrow‐sense heritability was calculated as:

(2) $${\hat{h}}^{2}=\;\frac{{{\hat{\sigma }}^{2}}_{a}}{{{\hat{\sigma }}^{2}}_{a}\;+\;{{\hat{\sigma }}^{2}}_{e}}$$

Standard errors of heritability estimates were obtained using the delta method implemented in the "vpredict" function of the ASReml‐R package. EBVs (estimated breeding values) for the ABLUP method and GEBVs (genomic‐estimated breeding values) for the GBLUP method were obtained from the best linear unbiased predictions (BLUPs) of the random additive effect (***a***).

To assess the similarity of the growth responses of the white spruce families to the different drought episodes at different ages that occurred between study sites, we estimated the Spearman rank correlation of family mean values between sites. Only the resistance component could be tested given the thinning at the Watford site following the drought episode of 2012 and its effects on subsequent growth, thus precluding the estimation of the postdrought components of drought response for this site.

### 2.8 Cross‐validation, predictive ability, and accuracy

The efficiency of GS models for predictions were evaluated using a cross‐validation (CV) approach. The offspring dataset was randomly split into 10 folds containing approximately 10% of the individuals of each family. For each round of CV, 9 of the 10 folds were used for model training, using the individual tree model at Equation 1, to predict the phenotypes and breeding values of the remaining fold. This procedure was repeated 10 times, for a total of 100 models run for each trait. The predictive ability (*PA*) was calculated as the Pearson correlation coefficient between the observed phenotypes (***y***) and the predicted pedigree‐based or genomic‐based breeding values obtained from cross‐validation models using ABLUP and GBLUP methods (***EBVs***, ***GEBVs***), respectively. Predictive accuracy (*PACC*) was also calculated as:

(3) $$PACC=PA/\sqrt{{\hat{h}}^{2}}$$

${\hat{h}}^{2}$ corresponding to the single‐trait heritability value obtained from Equation 2. To calculate *PACC* for both ABLUP and GBLUP methods, we used the ${\hat{h}}^{2}$ estimates obtained from the GBLUP method as our best estimates of the “true” heritability. Finally, a theoretical accuracy (${\hat{r}}_{i}$) was also calculated, not using the cross‐validation method above, but using the standard errors of the breeding values. The theoretical accuracy of the breeding value, for each individual and each trait, was calculated as:

(4) $${\hat{r}}_{i}=\;\sqrt{1‐{{\mathrm{SE}}^{2}}_{i}/\left(\left(1+F_{i}\right){{\hat{\sigma }}^{2}}_{a}\right)}$$

where *SE_i_* corresponds to the standard error of the breeding value of the i^th^ individual obtained from Equation 1 and *F_i_* is the inbreeding coefficient of the i^th^ individual. The value of *F_i_* was obtained using the diagonal elements of the *G* matrix, which are equal to 1+ *F_i_*. For each trait, the ${\hat{r}}_{i}$ values were averaged across all individuals to obtain a single mean $\hat{r}$ theoretical accuracy value.

### 2.9 Phenotypic and genetic correlations between traits

To assess correlations between components of drought response and DBH, height, acoustic velocity, and wood density, traits were paired one to each other in an identical bivariate model as the univariate one (Equation 1), as:

(5) $$\left[\begin{array}{c} y_{i}\\ y_{j} \end{array}\right]=\;X\beta (t)+Za(t)+\;e$$

where ***y_i_*** and ***y_j_*** correspond to a stacked vector of phenotypic values for traits *i* and *j*. ***β*** **(*t*)** is a vector of fixed effects including the mean for each trait and the block effect nested within trait. ***a*** **(*t*)** is the random additive genetic effect nested within trait, with **a(t)** ~ *N*(**0**, **V_a_** ⨂ **A**) for the ABLUP method, and ***e*** is the residual error, with **e** ~ *N*(**0**, ***V***_**e**_ ⨂ ***I***_**e**_). For the random additive genetic effect, the ***G*** matrix replaces the ***A*** matrix for the GBLUP method. The ***I***_**e**_ matrix is an identity matrix. The ***V***_**a**_ and ***V***_**e**_ matrices are 2 × 2 variance–covariance matrices defined by the correlation of effects between traits (${\hat{r}}_{a}$ and ${\hat{r}}_{e}$, respectively) and unique variances for each trait (CORGH in ASReml‐R). The genetic correlation between traits was directly obtained from the parameter ${\hat{r}}_{a}$, while phenotypic correlation (${\hat{r}}_{p}$) between traits was calculated as:

(6) $${\hat{r}}_{p(i,j)}=\;\frac{{\hat{\mathrm{COV}}\left(i,j\right)}_{p}}{\sqrt{{{\hat{\sigma }}^{2}}_{\mathrm{pi}}\;{{\hat{\sigma }}^{2}}_{\mathrm{pj}}\;}}=\;\frac{\;{\hat{r}}_{a}\sqrt{{{\hat{\sigma }}^{2}}_{\mathrm{ai}}\;{{\hat{\sigma }}^{2}}_{\mathrm{aj}}\;}+\;{\hat{r}}_{e}\sqrt{{{\hat{\sigma }}^{2}}_{\mathrm{ei}}\;{{\hat{\sigma }}^{2}}_{\mathrm{ej}}\;}}{\sqrt{\left(\;{{\hat{\sigma }}^{2}}_{\mathrm{ai}}+\;{{\hat{\sigma }}^{2}}_{\mathrm{ei}}\right)\left(\;{{\hat{\sigma }}^{2}}_{\mathrm{aj}}+\;{{\hat{\sigma }}^{2}}_{\mathrm{ej}}\right)\;}}$$

where ${\hat{\mathrm{COV}}\left(i,j\right)}_{p}$ is the estimated phenotypic covariance between the traits and ${{\hat{\sigma }}^{2}}_{\mathrm{pi}}$, ${{\hat{\sigma }}^{2}}_{\mathrm{ai}}$, ${{\hat{\sigma }}^{2}}_{\mathrm{ei}}$, ${{\hat{\sigma }}^{2}}_{\mathrm{pj}}$, ${{\hat{\sigma }}^{2}}_{\mathrm{aj}}$, which ${{\hat{\sigma }}^{2}}_{\mathrm{ej}}$ are the estimates of phenotypic, additive, and residual variance components for trait i and trait j, respectively. The significance of the genetic correlation was tested with a likelihood‐ratio test with one degree of freedom between the full model (Equation 5) and a reduced model assuming that ${\hat{r}}_{a}$ is equal to 0. The significance of the phenotypic correlation was tested with a likelihood‐ratio test with two degrees of freedom between the full model (Equation 5) and a reduced model assuming that ${\hat{r}}_{a}$ and ${\hat{r}}_{e}$ are all equal to 0.

### 2.10 Multi‐trait genetic selection schemes and genetic gains

We used as a reference the expected genetic gain that can be obtained by selecting for only one trait of interest (single‐trait selection) that was calculated as the mean of the top 5% estimated breeding values (EBVs, GEBVs) for that trait obtained from Equation 1. The estimated genetic gain in a context of multi‐trait selection was based on a selection index (SI) calculated as:

(7) $$SI=w_{1}*{\mathrm{DBH}}_{\mathrm{EBV}}+\;w_{2}*{\mathrm{Height}}_{\mathrm{EBV}}+\;w_{3}*Wood\;density_{\mathrm{EBV}}+\;w_{4}*Acoustic\;velocity_{\mathrm{EBV}}+\;w_{5}*{\mathrm{Recovery}}_{\mathrm{EBV}}+\;w_{6}*Relative\;resilience_{\mathrm{EBV}}+\;w_{7}*{\mathrm{Resilience}}_{\mathrm{EBV}}+\;w_{8}*{\mathrm{Resistance}}_{\mathrm{EBV}}$$

where ${\mathrm{DBH}}_{\mathrm{EBV}}$, ${\mathrm{Height}}_{\mathrm{EBV}}$, $Wood\;density_{\mathrm{EBV}}$, $ST300_{\mathrm{EBV}}$, ${\mathrm{Recovery}}_{\mathrm{EBV}}$, $Relative\;resilience_{\mathrm{EBV}}$, ${\mathrm{Resilience}}_{\mathrm{EBV}}$, and ${\mathrm{Resistance}}_{\mathrm{EBV}}$ are vectors of BLUP EBVs obtained from the ABLUP method for the corresponding trait and $w_{i}$ are the corresponding relative weight given to each trait. $w_{i}$ takes a value between 0 and 1 and $\sum_{i=1}^{8}w_{i}=1$. For the GBLUP method, GEBVs replaced EBVs. For each SI calculated, the corresponding genetic gain for each trait was calculated as the mean of the EBVs of the top 5% trees ranked according to SI. Since Quebec's white spruce genetic improvement program is highly focused on height growth, selection models chosen had a generally greater weight on this trait to respond to the objectives of the program. The other selection traits used were DBH, wood density, and acoustic velocity given that growth and wood quality traits are now generally assessed in most white spruce tests. A total of five multi‐trait selection scenarios were generated this way. The first scenario (S1) focused on height only with $w_{2}$ = 1 in the SI. The second (S2) and third (S3) selection scenarios, respectively, integrated the resistance and resilience components of drought response ($w_{i}$ = 0.2) alongside with height ($w_{2}$ = 0.8). Finally, the fourth and fifth selection scenarios, respectively, integrated resistance (S4) and resilience (S5), with $w_{i}$ = 0.2, but alongside with both height ($w_{2}$ = 0.6) and wood density ($w_{3}$ = 0.2). These scenarios allowed evaluating the impact, especially on height, of gradually integrating drought response as well as wood density in the scenarios while maintaining tree height as the top priority trait. For drought response, we chose to integrate only the resistance and resilience components in the calculation to constrain the number of scenarios tested.

## 3 RESULTS

### 3.1 Climate–growth relationships

**FIGURE 1:**
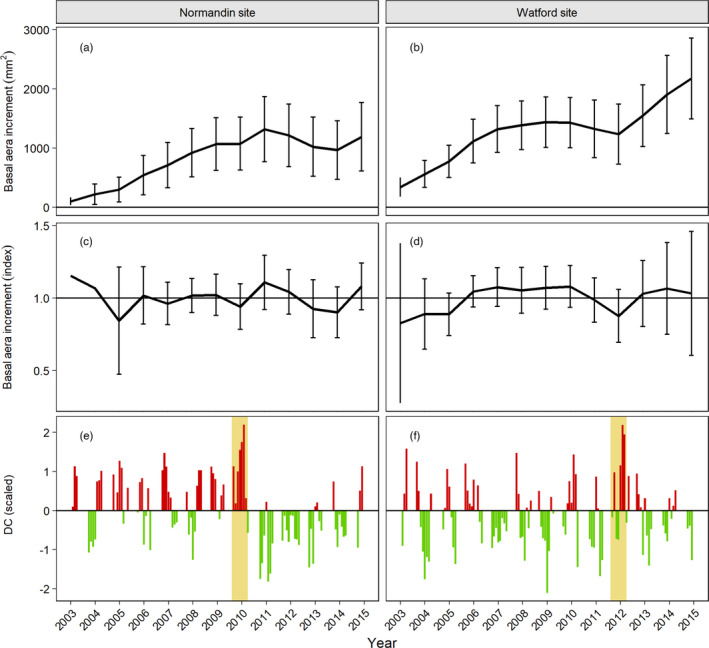
Mean annual basal area increment (BAI) indices from 2003 to 2015 for both Normandin (a) and Watford (b) study sites. Mean detrended BAI (index) for Normandin (c) and Watford (d). Mean chronologies were generated using the dplR package as described in the Material and Methods. Standard deviation of the means is presented by the error bars. Standard deviation of the index mean for the years 2003 and 2004 at the Normandin site is not presented for a better visualization. Scaled monthly drought code (DC) is presented for the Normandin site (e) and for the Watford site (f) for this period. Regarding the scaled DC values, the red color corresponds to drier than usual conditions (positive scaled DC), the green color wetter than usual conditions (negative scaled DC). The position of the year on the x‐axis corresponds to the separation between the months of June and July

To evaluate the drought stress response of trees, simulated climatic data and radial growth were first compared to determine whether dry conditions had taken place and had impacted tree growth on each of the two experimental sites. Sites raw and mean detrended growth values are presented in Figure 1 for both sites alongside scaled drought code (DC) values. For the Normandin site, 2010 presented higher than usual DC values for much of the growing season from May to September. These dry conditions were matching a radial growth slowdown the same year even if trees were still much in their juvenile phase of yearly increase of BAI. Therefore, 2010 was the reference year for which all four drought response traits were calculated to estimate their heritabilities at this site. For the Watford site, dry conditions were noted in 2012 but later in the growing season from July to September, with a more pronounced growth slowdown coupled to dry conditions, hence corresponding to more severe drought effects than those observed at the Normandin site in 2010. This growth slowdown was observed after BAI at this site had reached a plateau, indicating that stand closure was reached earlier at this site characterized by more favorable growth conditions than those at Normandin. Therefore, for Watford, the resistance component of drought response was calculated based on the drought stress signature recorded in 2012. However, given stand closure, thinning of the Watford test plantation was conducted in the fall of 2012 after the growing season. Because the drought and thinning effects could not be disentangled in the trees’ response traits following the drought episode, for this site we could only estimate the resistance component of drought response corresponding to the growth reduction of the current year (Lloret et al., 2011), and did not consider the postdrought period that corresponded to the post‐thinning period.

**FIGURE 2:**
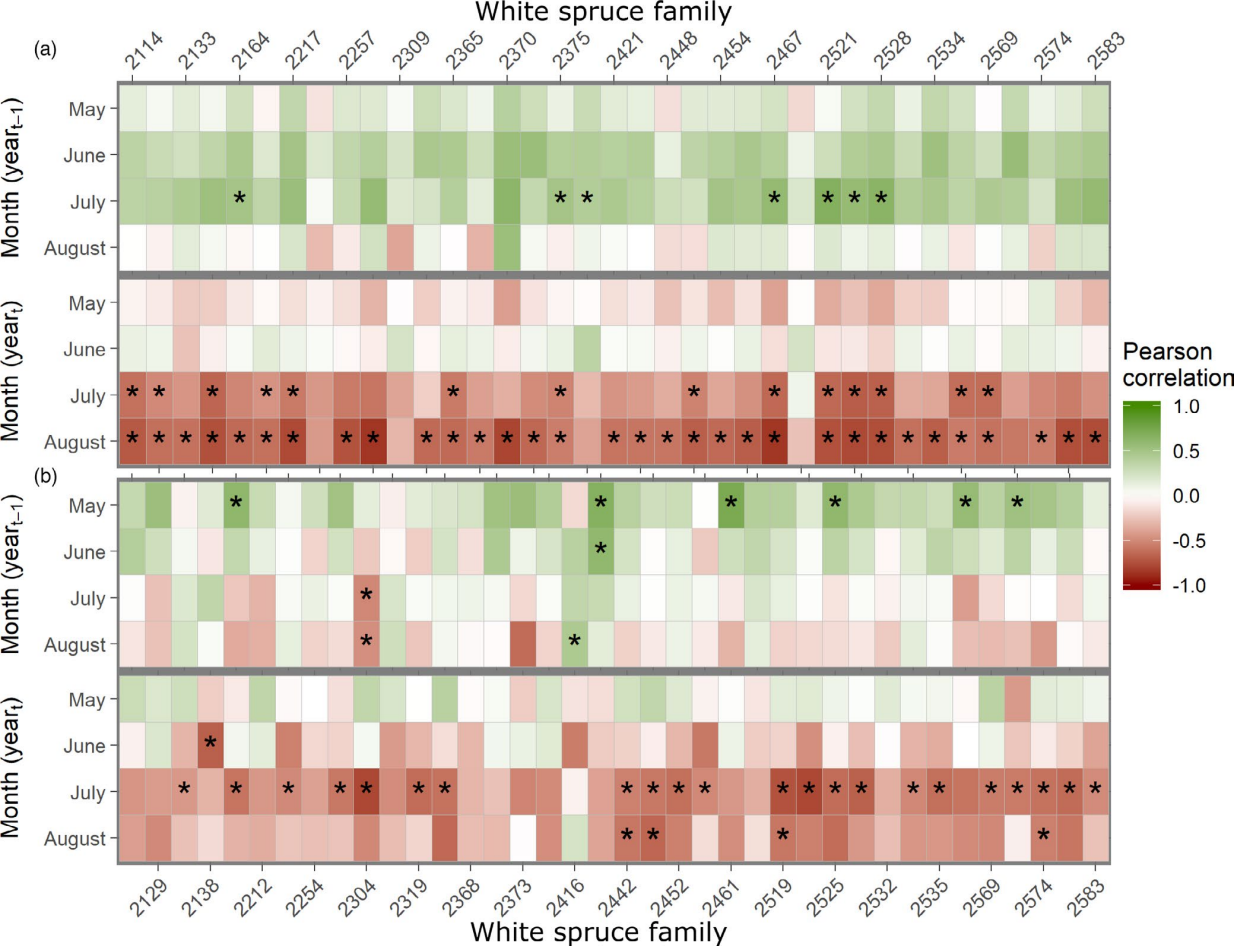
Pearson correlations between mean family basal area increment (BAI) indices and monthly drought code (DC) for the Normandin (a) and Watford (b) study sites. Families are presented on x‐axis, months on y‐axis. The preceding year months appear on the upper half, and the current year months, on the lower half. Significant correlations (*p* < 0.05) as calculated with the “dcc” function of the treeclim R package are shown by an asterisk

To analyze the response of radial growth to variation in water availability throughout the tree lifespan and therefore, ensure that observed growth reductions were indeed related to dry conditions, mean family BAI between 2003 and 2015 was correlated to previous and current year's months of May, June, July, and August DC values for each study site using a bootstrap approach. These climate–growth relationships were estimated at the family level. The mean detrended family chronologies at each site are provided in Figure S2. Correlations between family growth chronologies and site DC values for current and previous years are presented in Figure 2 for both Normandin and Watford study sites. Normandin's heatmap showed several negative and significant correlations between families BAI and DC (and SMI) in July and August of the current growing season for multiple families, indicating that water availability was a limiting factor to radial growth at this site for much of the growing season during the tree lifespan. At the Watford site, significant correlations between BAI and DC (and SMI) were observed for the current month of July but less for August. These large and significant correlations at the family level are in accordance with those found at the site level (Table S2). These results indicate that the effect of limited water availability on current growth was generally delayed at Normandin compared to Watford during the tree lifespan. There was also variation among families in climate–growth relationships. For example, there were nonsignificant and lower correlation values for these months for many families at both sites. These trends suggest the presence of genetic differentiation in radial growth and relationships with climate that will be further investigated below with statistical models.

### 3.2 Correlations between drought response traits and conventional traits

Phenotypic and genetic correlations between components of drought response and conventional traits were investigated separately for each study site given the different drought episodes experienced on different years and stages of maturity of the trees and stand closure, and differences in the timing, duration, and amplitude of drought effects on BAI. The genetic and phenotypic correlations between drought response components and conventional traits using GBLUP are presented in Table 2 (see Tables S3 to S6 for all correlations for both ABLUP and GBLUP methods). For the Normandin site, the number of significant phenotypic correlations was larger than that for genetic correlations, but their values were generally low. The recovery and relative resilience components of drought response had positive and significant phenotypic correlations with growth traits (tree height, DBH, and EW area). On the other hand, the resistance component of drought response had negative phenotypic correlations with growth traits (height, DBH, and EW and LW area). Genetic correlations were also generally low (under 0.30) and rarely significant. Only two significant genetic correlations were detected at the Normandin site. LW area had significant negative correlations with the resilience (−0.55) and the resistance (−0.66) components of drought response.

**TABLE 2:** Correlations between drought response traits and conventional growth and wood traits^a^ using GBLUP^b^. Results for the Normandin study site are presented in the upper half of the table (a) and those for the Watford study site as the lower half (b). For each site, genetic correlations are above the center line and phenotypic correlations below it. Standard errors are shown in parentheses along with levels of statistical significance indicated by asterisks^c^

Traits^a^	Height	DBH	Acoustic velocity	Wood density	EW density	LW density	EW area	LW area	Recovery	Relative resilience	Resilience	Resistance
(a) Normandin site												
Recovery	0.41 (0.24)	0.22 (0.27)	−0.18 (0.29)	−0.32 (0.27)	−0.23 (0.27)	0.36 (0.39)	0.20 (0.29)	−0.12 (0.31)	‐			
Relative resilience	0.44 (0.24)	0.18 (0.27)	−0.15 (0.28)	−0.37 (0.25)	−0.28 (0.26)	0.29 (0.39)	0.16 (0.30)	−0.17 (0.31)	0.99^†^	‐		
Resilience	0.35 (0.26)	−0.14 (0.30)	−0.09 (0.30)	−0.42 (0.26)	−0.31 (0.26)	−0.05 (0.40)	−0.13 (0.32)	−0.55* (0.28)	0.79** (0.15)	0.85** (0.11)	‐	
Resistance	0.04 (0.33)	−0.49 (0.27)	0.07 (0.33)	−0.31 (0.32)	−0.21 (0.31)	−0.54 (0.40)	−0.47 (0.30)	−0.66** (0.23)	−0.06 (0.39)	0.06 (0.39)	0.58 (0.26)	‐
Recovery	0.19** (0.06)	0.18** (0.06)	−0.02 (0.06)	−0.17** (0.06)	−0.13 (0.06)	0.02 (0.06)	0.16** (0.06)	0.05 (0.06)	‐			
Relative resilience	0.16** (0.06)	0.13* (0.06)	−0.04 (0.06)	−0.15* (0.06)	−0.11 (0.06)	0.02 (0.06)	0.12 (0.06)	0.01 (0.07)	0.97^†^ (0.00)	‐		
Resilience	0.04 (0.07)	−0.08 (0.06)	−0.03 (0.06)	−0.12 (0.06)	−0.09 (0.06)	0.01 (0.06)	−0.04 (0.06)	−0.13 (0.06)	0.72*** (0.03)	0.81*** (0.02)	‐	
Resistance	−0.15** (0.06)	−0.31*** (0.06)	0.00 (0.06)	0.00 (0.06)	−0.01 (0.06)	−0.02 (0.06)	−0.22** (0.06)	−0.24** (0.06)	−0.05 (0.06)	0.06 (0.06)	0.64*** (0.04)	‐
(b) Watford site												
Resistance	0.59** (0.19)	0.45 (0.25)	0.05 (0.26)	0.18 (0.27)	0.24 (0.27)	0.13 (0.29)	0.40 (0.28)	0.34 (0.27)				
Resistance	0.49*** (0.05)	0.49*** (0.05)	0.12* (0.07)	−0.05 (0.07)	−0.03 (0.07)	0.10 (0.07)	0.41*** (0.05)	0.33*** (0.06)				

^a^ See Table 1 for a full description of traits.

^b^ The model fitted for each combination of traits is described in Equation 5.

^c^ Levels of statistical significance: * *p* < 0.05, ** *p* < 0.01, *** *p* < 0.001, ^†^ convergence failed.

For the Watford site, given the thinning of the test in the fall of 2012 and its effect on subsequent growth, we could only estimate the correlations implicating the resistance component of drought response occurring during the 2012 growing season when a drought episode occurred. At this site, resistance had a positive significant genetic correlation with height (0.59) and positive phenotypic correlations with all growth traits (height, DBH, and EW and LW areas) (Table 2). Thus, opposite significant phenotypic correlations were observed between the resistance component of drought response and growth traits between the two study sites. These opposite correlations were not observed at the genetic level, thus not impacting differently the outcomes of multi‐trait selection schemes between sites (see below).

### 3.3 Different growth responses of white spruce families to the different drought episodes

Given that drought episodes differed between sites in their year of occurrence and severity of effects on BAI, and given the different stages of maturity of the trees and stand closure, we considered sites separately in the statistical modeling analyses and relied on a Spearman rank correlation of families’ values between sites to estimate the similarity in the growth responses of the white spruce families to the different drought episodes affecting the study sites at different ages. The rank correlation was low (0.10) and nonsignificant for the resistance component of drought response, indicating that families reacted differently from site to site affected by different drought episodes at different ages of the trees. This result is in line with the opposite phenotypic correlations observed between sites between the resistance component of drought response and the cumulative growth traits, which was not observed for genetic correlations.

### 3.4 Heritability estimates

To assess the genetic control of drought response traits, the additive genetic effect component from individual tree models was used to estimate narrow‐sense heritability values for each trait. Heritability estimates are presented for both sites in Table 3. A summary of estimates of the variance components for all traits is also presented in Table S7. For both sites, we found significant additive genetic effects for drought response components, but their heritability estimates (${\hat{h}}^{2}$) were generally lower than those for conventional growth and wood quality traits. At both sites, there was no systematic differences observed between pedigree‐based ABLUP and genomic‐based GBLUP. However, standard errors of the estimates obtained from GBLUP were always equal to or lower than those obtained from ABLUP, even if a marker‐informed and thus, fully validated pedigree was used for ABLUP.

**TABLE 3:** Individual narrow‐sense heritability estimates^a^ (${\hat{h}}^{2}$) for drought response traits and conventional growth and wood traits for both Normandin and Watford study sites obtained with ABLUP and GBLUP^b^. Standard errors around heritability estimates are shown in parentheses. Levels of statistical significance^c^ shown correspond to that of the additive genetic variance component (${{\hat{\sigma }}^{2}}_{a}$)

Traits^d^	Narrow‐sense heritabilities			
	Normandin site		Watford site	
	ABLUP	GBLUP	ABLUP	GBLUP
Recovery	0.29 (0.12)***	0.22 (0.10)***	‐	‐
Relative resilience	0.29 (0.12)***	0.23 (0.10)***	‐	‐
Resilience	0.24 (0.11)**	0.20 (0.10)**	‐	‐
Resistance	0.13 (0.10)*	0.16 (0.10)*	0.22 (0.11)**	0.25 (0.11)**
Height	0.44 (0.14)***	0.47 (0.12)***	0.45 (0.13)***	0.46 (0.12)***
DBH	0.44 (0.14)***	0.45 (0.12)***	0.31 (0.13)**	0.32 (0.12)***
Acoustic velocity	0.46 (0.14)***	0.41 (0.12)***	0.64 (0.14)***	0.60 (0.11)***
Wood density	0.44 (0.14)***	0.42 (0.12)***	0.48 (0.14)***	0.41 (0.12)***
EW density	0.50 (0.14)***	0.44 (0.12)***	0.47 (0.14)***	0.41 (0.12)***
LW density	0.13 (0.10)	0.16 (0.10)*	0.33 (0.13)***	0.42 (0.12)***
EW area	0.37 (0.14)***	0.37 (0.12)***	0.25 (0.12)**	0.28 (0.11)***
LW area	0.36 (0.13)***	0.46 (0.13)***	0.31 (0.12)***	0.33 (0.11)***

^a^ Narrow‐sense heritability was calculated from Equation 2.

^b^ The model fitted is described in Equation 1.

^c^ Levels of statistical significance: * *p* < 0.05, ** *p* < 0.01, *** *p* < 0.001.

^d^ See Table 1 for a full description of traits.

### 3.5 Predictive ability and accuracy of GBLUP and ABLUP

**FIGURE 3:**
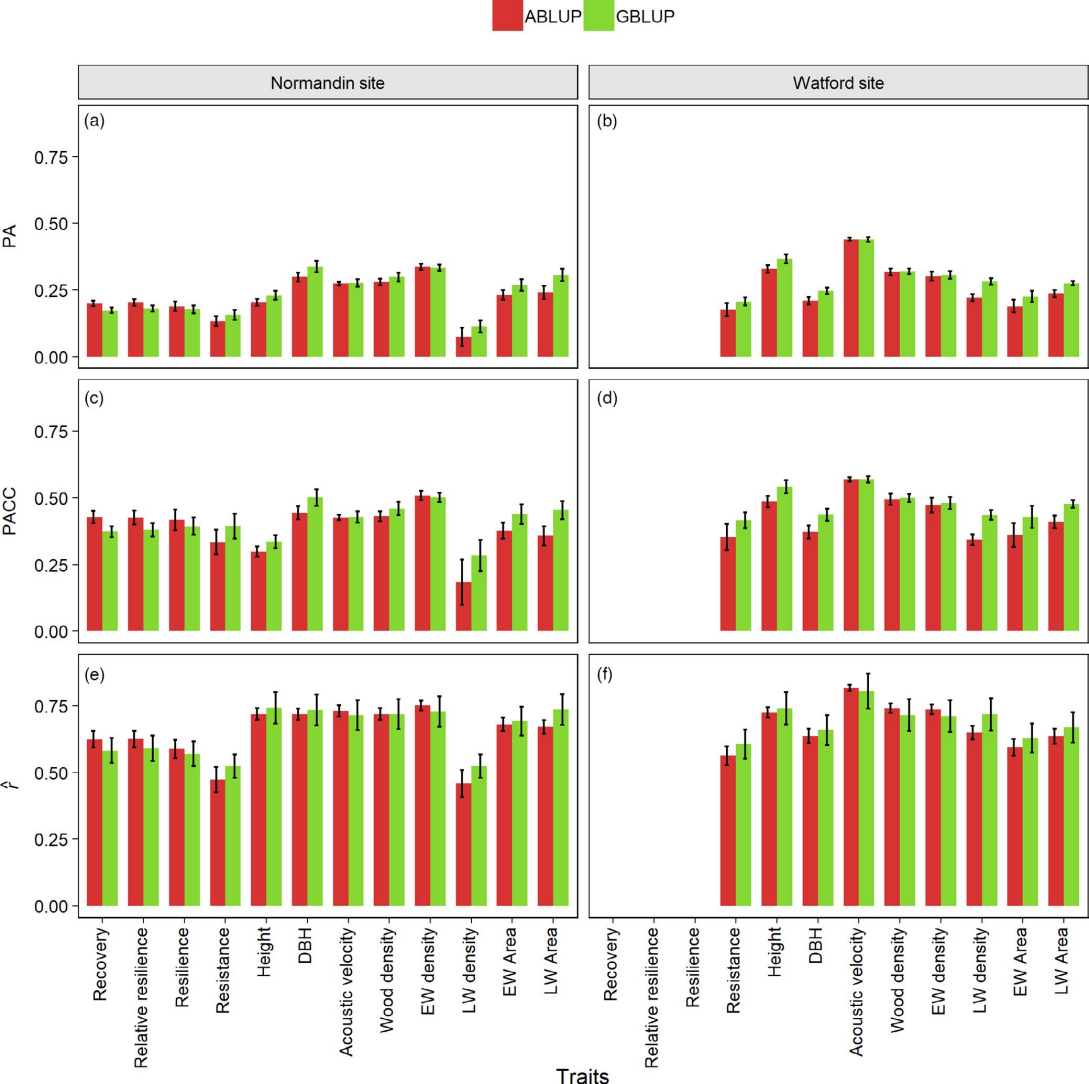
Predictive ability (PA), predictive accuracy (PACC), and theoretical accuracy ($\hat{r}$) for each trait measured at the Normandin study site (respectively (a), (c), and (e)) and the Watford study site (respectively (b), (d), and (f)). Error bars on the histograms represent standard deviations. The drought response trait components recovery, resilience, and relative resilience could not be calculated for the Watford site due to a plantation thinning right after the drought episode of 2012. See section “Cross‐validation, predictive ability and accuracy” of Materials and Methods for calculation methods

Predictive ability (PA) and predictive accuracy (PACC) for each trait were estimated for both pedigree‐based ABLUP and genomic‐based GBLUP models using a cross‐validation approach. Theoretical accuracy ($\hat{r}$) of the estimated breeding values was also estimated for each trait and both ABLUP and GBLUP models. These values are presented in Figure 3. ABLUP and GBLUP resulted in similar accuracy estimates for both study sites. All three accuracy estimators had values for drought response traits that were slightly lower than those obtained for conventional growth and wood quality traits. The differences were less notable for PACC, and $\hat{r}$ than for PA. These differences reflected to a large degree those seen in heritability estimates.

### 3.6 Genetic gain and selection schemes

To investigate the effect of selecting for conventional traits on drought response traits and integrating those in selection schemes, genetic gains for individual traits were estimated as well as for several multi‐trait selection scenarios. They are presented in Table 4. Genetic gains estimated for conventional traits showed no apparent differences between the ABLUP and GBLUP methods. For the Normandin site and using GBLUP, the genetic gains estimated from single‐trait selection for the conventional traits (DBH, height, wood density, and acoustic velocity) varied from 7.4% for wood density to 19.2% for DBH. For the relative resilience component of drought response, given that the genetic gain was estimated as a percentage of the phenotypic mean and that the phenotypic mean was small (see Table 1), an apparent higher genetic gain of 136.3% was obtained. Given its scaling, the amplitude of the genetic gain estimated for this trait must be interpreted with caution in the context of tree improvement. However, the important coefficient of variation for this trait (Table 1) has also contributed positively to this gain estimate. The genetic gains from single‐trait selection for the other drought response traits varied from 16.7% for the resilience component to 7.4% for the resistance component of drought response. Among all multi‐trait scenarios tested, scenarios S4 and S5, which integrated wood density, were the only ones showing positive genetic gains for every trait including for all components of drought response, while generating a near maximal gain for height with both ABLUP and GBLUP methods. For the Normandin site, scenario S5 (selecting for height at 0.6, wood density at 0.2, and the resilience component of drought response at 0.2) resulted in higher genetic gains for DBH, as well as for the recovery and resilience components of drought response with both ABLUP and GBLUP methods compared to scenario S4, which prioritized the resistance component of drought response.

**TABLE 4:** Estimated genetic gains when selecting the 5% top individuals from single‐trait selection and for five multi‐trait index selection scenarios (S1 to S5) based on individual breeding values (BVs) for the Normandin (a) and Watford (b) study sites. Selection traits are DBH, tree height, wood density, acoustic velocity, and the four drought response components, recovery, relative resilience, resilience, and resistance. Genetic gain values are expressed as a percentage of the mean of the trait. Fractions below multi‐trait selection scenarios indicate the relative weight of priority traits included in the index of each scenario

Selection methods and scenarios^a^, ^b^, ^c^	DBH	Height	Wood density	Acoustic velocity	Recovery	Relative resilience^d^	Resilience	Resistance
(a) Normandin site	Estimated genetic gains (%)^c^							
ABLUP								
Single‐trait selection	19.3	12.5	7.8	11.9	16.3	171.2	19.9	6.6
S1 (height = 1)	7.3	12.5	−0.5	1.9	1.7	14.0	1.0	−0.1
S2 (height = 0.8, resistance = 0.2)	6.7	12.3	−0.6	2.4	1.3	14.1	2.8	1.4
S3 (height = 0.8, resilience = 0.2)	6.9	12.0	−0.7	1.0	4.4	44.7	5.8	1.7
S4 (height = 0.6, wood density = 0.2, resistance = 0.2)	5.2	12.2	0.6	3.3	1.5	17.0	3.5	1.7
S5 (height = 0.6, wood density = 0.2, resilience = 0.2)	6.6	11.9	0.6	2.8	3.9	39.4	5.5	1.7
GBLUP								
Single‐trait selection	19.2	12.9	7.4	11.2	12.4	136.3	16.7	7.4
S1 (height = 1)	9.4	12.9	−1.0	−0.2	1.4	11.1	−0.3	−1.1
S2 (height = 0.8, resistance = 0.2)	7.5	12.5	−1.0	0.9	1.4	15.2	2.3	1.0
S3 (height = 0.8, resilience = 0.2)	7.9	12.4	−1.0	0.6	2.4	24.4	3.3	1.1
S4 (height = 0.6, wood density = 0.2, resistance = 0.2)	6.5	12.2	1.3	2.5	0.9	11.0	1.7	0.8
S5 (height = 0.6, wood density = 0.2, resilience = 0.2)	7.5	12.3	0.4	2.3	2.4	24.7	3.3	1.1
(b) Watford site	Estimated genetic gains (%)							
ABLUP								
Single‐trait selection	8.6	8.5	8.7	14.0				12.7
S1 (height = 1)	6.7	8.5	−0.3	1.1				3.0
S2 (height = 0.8, resistance = 0.2)	7.0	7.7	−1.2	1.4				8.3
S4 (height = 0.6, wood density = 0.2, resistance = 0.2)	5.0	7.4	2.4	3.9				7.4
GBLUP								
Single‐trait selection	10.0	10.0	7.5	13.9				14.7
S1 (height = 1)	7.9	10.0	0.1	1.9				3.8
S2 (height = 0.8, resistance = 0.2)	8.0	9.3	−0.1	1.2				9.2
S4 (height = 0.6, wood density =0.2, resistance = 0.2)	7.2	8.6	0.8	1.6				10.8

^a^ The model fitted for each trait to calculate breeding values is described in Equation 1.

^b^ The index selection formula is presented in Equation 7. Numbers in parentheses indicate trait weights.

^c^ See Table 1 for a full description of traits.

^d^ A large relative genetic gain was obtained given that the average value for this trait was close to 0.

For the Watford site, genetic gains estimated from single‐trait selection varied from 7.5% for wood density to 13.9% for acoustic velocity. The resistance component of drought response showed the highest genetic gain with 14.7% using GBLUP, while acoustic velocity showed the highest maximum gain (14.0%) using ABLUP. Positive genetic gains were also observed for all traits at the Watford site when using S4. Compared to S1, which focused only on height, S4 showed a little decrease in gain for DBH and height, but it resulted in a considerable gain for the resistance component of drought response, especially with GBLUP.

## 4 DISCUSSION

### 4.1 Factors affecting drought response

Using a bootstrapped correlation approach spanning the 2003–2015 period, we found that both study sites were limited at time by the current summer water availability with noticeable negative impacts on radial growth during the tree lifespan. Such an approach was previously used to detect relationships between drought stress and growth reductions during the lifespan of white spruce trees (Chen et al., 2017; Depardieu et al., 2020). Because both study sites suffered growth reductions synchronized with maxima DC values, our results indicate that water availability was a main driver of radial growth, with growth reductions likely representing physiological stress signatures resulting from limited water availability.

Our results also suggest that water availability was not as much a general limiting factor at the Normandin site during the 2010 growing season than it was at the Watford site in 2012, in spite of a longer drought episode lasting from July to August but with lesser growth reduction effects (Figure 1). Also, family ranks with respect to the resistance component of drought response between sites were poorly conserved, likely linked to the different age and severity of effects of drought episodes between sites. It has already been suggested that the fluctuations in drought responses between individuals may be more closely related to differences in the intensity of drought episodes than to genetic variation (Lloret et al., 2011). Opposite phenotypic correlations were also noted between sites, between the resistance component of drought response and growth traits. At Watford, these correlations were positive, as expected and as previously observed for white spruce trees affected by a severe drought episode at an older age (Depardieu et al., 2021), indicating that trees with more vigorous growth during their lifespan resisted better during the current year of a drought episode having more severe effects on BAI. It could be that these trees at the time of drought had developed better root systems, allowing them to better sustain severe drought stress by limiting the damages from fine root loss (Gaul et al., 2008).

Contrary to this finding and those of Depardieu et al. (2021), trade‐offs have been frequently proposed or reported between growth traits and resistance to abiotic stress, such as drought. It was reported that lower lumen diameter resulting from lower cell and xylem expansion (Bowyer et al., 2005) might lead to greater resistance to cavitation during drought episodes (Tyree & Zimmermann, 2002). Trade‐offs between growth and resistance to climatic stresses such as cold temperatures have also been observed in white spruce (Sebastioan‐Azcona et al., 2018), while also reported for both drought and cold stresses in Douglas‐fir (*Pseudotsuga menziesii*) (Darychuck et al., 2012). Also, wood density has been reported as a possible screening trait for drought resistance (Rosner et al., 2014) although in our study, genetic correlations between these traits were not significant. However, it was reported that the phenotypic variation in embolism resistance resulting from cavitation was lower than for growth and xylem conductivity (Gonzalez‐Munoz et al., 2018), indicating that phenotypic variation for drought response might be related to other traits not assessed in our study, such as water‐use efficiency, stomatal conductance, or leaf area (Aubin et al., 2016).

At the Normandin study site where a longer drought episode with lesser effects on BAI was noted in 2010, significant positive correlations were noted between cumulative growth traits and the recovery and relative resilience components of drought response, in agreement with an earlier study of a more mature white spruce provenance/progeny test suffering from a severe drought (Depardieu et al., 2021). However, for the resistance component of drought response, a surprisingly opposite pattern was observed to that observed at the Watford study site and in Depardieu et al. (2021), with low but significant negative phenotypic correlations with cumulative growth traits. Whether the longer duration and earlier timing of the drought episode at Normandin but with lesser impact on current growth could explain this opposite pattern remains uncertain. This apparent incongruity could also be linked to several differences in environmental and developmental factors entangled with differences in drought episodes between sites, and each potentially impacting the severity of drought effects. For instance, the drought episode occurred 2 years earlier in Normandin than in Watford, at a time when 15‐year‐old trees were more in their juvenile phase of increasing BAI (Figure 1), with possible different physiological response to water stress and less severe effects of limited water availability on current year growth than that observed at Watford 2 years later with a different drought episode. Indeed, at Watford, trees’ BAI reached a plateau a few years before the 2012 drought episode, at the time when trees were closer to their transition from their juvenile to mature stage (Lenz et al., 2010). Thus, this apparent difference in the maturity of the trees between sites at the time of drought episodes might have influenced the severity of drought effects and impacted trees’ drought resistance component differently.

Also, because the Watford more southerly study site was characterized by more favorable growth conditions due to milder climate than that at Normandin, crown closure was more complete at Watford at the time of the 2012 drought episode than at Normandin at the time of the 2010 drought episode. This larger stand closure even necessitated the thinning of the plantation during the fall of 2012 with ensuing growth release during the following years (see Figure 1), while preventing us to evaluate at this site the other components of drought response based on postdrought growth patterns. Thus, higher stand density had likely resulted in more competition effects at Watford during the shorter drought episode of 2012 than at Normandin during the longer drought episode of 2010. The moisture regime could have been modified with water availability being more limited during the drought episode (Bottero et al., 2017; Clark et al., 2016; Gleason et al., 2017), a pattern which was reflected in more severe effects on BAI at this site. Given that growth sensitivity to drought has been reported to vary with soil moisture regime (Griesbauer et al., 2021), that tree root systems could be more highly susceptible to embolism during severe drought stress (Domec et al., 2004), these factors might have contributed to more detrimental effects on current growth at Watford during the shorter drought episode of 2012 than at Normandin during the longer drought episode of 2010. Thus, all evidence points at more severe drought effects at the Watford study site in 2012 than at the Normandin site in 2010.

These results, thus, constrained us to consider analyses at the site level given the different timing and intensity of the drought stress between sites, together with other site‐specific environmental or developmental factors discussed above. These various factors further indicate that the analysis of the genetic and environmental components of tree's response to drought stress must be carefully analyzed at different ages of the trees and on different sites, and whenever possible by considering different drought episodes ideally repeated across sites. Our results indicate the complexity of studying the genetic control of drought response traits over different environments and drought episodes, even more than drought response traits are based on measurement of single year responses contrary to cumulative growth and wood quality traits. Thus, growth declines and recoveries observed during a few intense drought periods spread over different ages and sites might represent the ideal, yet difficult to obtain, conditions to select more accurately the most favorable genotypes for drought resistance. This is especially so when a drought episode is geographically widespread, thus affecting different sites in a more similar manner, notwithstanding site‐specific conditions that may affect soil moisture and water availability. The effects of such a severe drought episode on older half‐sib families of white spruce were previously assessed (Depardieu et al., 2020, 2021). The patterns that they observed for the resistance component of drought response were more in line with those observed in this study for the Watford site affected by more severe drought effects, where resistance was positively correlated to traits related to lifespan tree vigor. While this study did not aim at specifically quantifying the phenotypic response of growth to drought stress conditions, the results obtained here raise the need for caution when estimating the components of drought response. This is especially true with younger material that is still fluctuating in radial growth trends annually. In such case, use of analytical strategies assuming homogeneity of drought stress effects among drought episodes, sites or developmental stages of the material tested should be avoided. We should also stress that selections for improved drought resistance should probably be constrained within breeding zones, such as those defined for white spruce in the province of Québec (Li et al., 1997) in order to maintain other aspects of tree adaptation.

### 4.2 Genetic control of drought response traits

Heritability estimates for drought response traits assessed on one site or the other were significant, indicating the existence of significant natural genetic variation among polycross families for potential use by tree breeding. This result is line with those of a previous study on a large provenance/progeny white spruce test, where narrow‐sense heritability estimates could be estimated from open‐pollinated families raised in a different site and assessed after a severe drought episode at a later age (Depardieu et al., 2020). At the Normandin site, the most precise heritability estimates obtained using GBLUP were marginally lower for the recovery, relative resilience, and resilience components of drought response (${\hat{h}}^{2}$ of 0.22, 0.23, and 0.20, respectively), compared to conventional traits related to cumulative growth and wood traits, and the trend was the same with ABLUP. For the resistance component of drought response which could be assessed for both sites and drought episodes, significant genetic variance was observed for both episodes, but heritability was also marginally lower compared to conventional traits. Higher heritability estimates are often obtained using ABLUP (Beaulieu et al., 2020) but in our study, we corrected ABLUP for pedigree errors using genomic profiles, thus making heritability estimates from ABLUP more accurate and comparable to those obtained with GBLUP. More accurate estimates of relatedness between individuals should also be obtained with GBLUP, which takes into account Mendelian sampling contrary to ABLUP (de Almeida Filho et al., 2019).

The heritability estimates obtained in this study were higher for tree height, DBH, wood density, and acoustic velocity than those previously reported by Lenz, Nadeau, Azaiez, et al. (2020). These authors relied on the complete polycross test replicated on both the Normandin and Watford study sites, but also on a third site, Valcartier (N46°58’, W 71°28’), which was not affected by a drought episode and thus, could not be considered in the present study. The inclusion of the genotype‐by‐environment interaction effect (*GxE*) in their statistical models can largely explain the differences in heritability estimates obtained for conventional traits, which was not possible in this study because of the different impact of the drought episodes on current growth and several other environmental and developmental stage differences observed between the Normandin and Watford sites at the time of drought episodes. In their study, Lenz, Nadeau, Azaiez, et al. (2020) estimated that *GxE* was moderate for growth traits, such as height and DBH (type‐B correlations between 0.60 and 0.70), but lower for wood quality traits, such as acoustic velocity and wood density (type‐B correlations between 0.92 and 1), as usually observed for white spruce and other conifers (Beaulieu et al., 2014). Using a Spearman rank correlation of family means, ranks were not conserved between sites for the resistance component of drought response, in relation to drought episodes of different severity of effect on current growth, as well as different environmental conditions and developmental stage of the genetic material at the time of drought episodes between sites. This result indicates that, on average, families reacted differently to these drought episodes of different severity of effects, echoing the opposite phenotypic correlations observed between sites between the resistance component of drought response and cumulative growth traits.

For both sites and drought episodes, marginally lower heritability estimates were obtained for drought response traits, compared to conventional cumulative growth and wood traits, which are the result from trait expression over tree lifespan. Among these, the resistance component was the least heritable at the Normandin site where the four components of drought response could be estimated (see Table 2). This result conforms with the observations of Depardieu et al. (2020) where the resistance component was also the least heritable drought stress response traits in a mature white spruce provenance/progeny test having experienced a drought at a later age. However, heritability was higher for the resistance component of drought response at the Watford site where more severe drought effects on current growth were observed, which indicates that likely more genetic gains could be obtained under severe drought effects. Beside this study and that of Depardieu et al. (2020), to our knowledge, the quantitative genetic control for drought response traits as defined by Lloret et al. (2011) has not been analyzed in any other conifer species, especially in the context of genomic selection. Overall, the significant genetic control for all drought response traits that we studied suggests that there is potential for genetic improvement, as discussed below.

### 4.3 Predictive accuracies of GS models

While high, the predictive ability and accuracy of GS and pedigree‐based predictive models were generally lower for the components of drought stress response than for growth and wood quality traits. This trend was expected given the marginally lower heritability for drought response traits compared to conventional traits. However, using the same cross‐validation approach in a combined‐site analysis of the same white spruce test, Lenz, Nadeau, Azaiez, et al. (2020) reported slightly higher PA and PACC values for the traits tree height, DBH, wood density, and acoustic velocity. It is, thus, likely that the reduced number of trees used in our single‐site analyses, 281 at the Normandin site and 279 at the Watford site, compared to the 856 trees of Lenz, Nadeau, Azaiez, et al. (2020) for their combined‐site analysis that included a third site not affected by drought episodes, explain in a large part the lower model accuracies obtained in the present study. As explained above, we chose not to include a "site" effect in the statistical model due to the different impacts and contexts of the drought episodes between sites, and therefore, use a cautious analytical approach considering the two sites separately.

The use of GBLUP instead of ABLUP did not cause a uniform improvement in accuracy whether PA, PACC, or $\hat{r}$ were considered. ABLUP and GBLUP resulted in very similar accuracy values for all traits. High congruence between accuracy estimates derived from the two methods was expected, given that for ABLUP, pedigree information was validated and paternal contributions were recovered from marker information. This approach was necessary to recover both parents in a half‐sib mating design, such as polycross mating. However, more biases from ABLUP are expected when, for example, a mating design is used where both parents are theoretical, and none is recovered from marker information. However, regarding heritability and estimated breeding values using ABLUP, Vidal et al. (2015) observed only a small bias while working without paternity recovery in two maritime pine (*Pinus pinaster* Ait.) polycross tests.

### 4.4 Genetic correlations and indirect selection

Genetic correlations are useful to tree breeders in order to detect adverse effects of selecting for one trait on other traits and use of multi‐trait selection schemes to counterbalance these effects. For the components of drought response, no significant adverse genetic correlations nor trade‐offs with cumulative growth or wood quality traits were observed other than those between the resilience and resistance components of drought response and latewood basal area for the Normandin site. However, for both study sites, adverse genetic correlations between growth and wood quality traits were observed (see the full correlation tables in Tables S3 to S6 for both sites and for both ABLUP and GBLUP), as previously observed in white spruce and other conifers (Lenz et al., 2013). These correlations relate well with the reported results of increased tracheid diameter and length with decreased cell wall thickness (Bowyer et al., 2005). Therefore, in order to improve for both tree growth and wood quality, the consideration of a multi‐trait selection scheme involving wood density should be adequate to counterbalance the negative effect on wood quality, of selecting for tree height only (Lenz, Nadeau, Azaiez, et al., 2020).

Genetic correlations are also useful to examine the feasibility of indirect selection for a trait by selecting for another trait that is less time‐consuming to assess. Such an example is when selecting for a trait requesting a large phenotyping effort, such as natural pest resistance (Lenz, Nadeau, Mottet, et al., 2020) or in the present case, drought response traits. In this study and for both study sites and drought episodes, genetic correlations were generally positive between the component of drought response and tree height and to a lesser extent DBH, in accordance with a previous study of a more mature provenance–progeny test of white spruce experiencing an intense drought episode (Depardieu et al., 2021). But due to the present site‐by‐site analysis, sampling sizes were not large enough for the correlations to reach statistical significance in many instances. However, these results are encouraging and suggest that selection for tree height, a targeted trait for white spruce, might have no significant negative impact on components of drought response and in some cases, a positive impact. For instance, for the Watford study site, where more severe effects of drought stress on BAI were observed, a significant positive genetic correlation was obtained between the resistance component of drought response and tree height, in support of similar observation using more mature white spruce genetic material (Depardieu et al., 2021). As noted before from phenotypic correlations, this correlation indicates that genetically more vigorous trees will generally better resist a severe water stress and that indirect selection for resistance to severe drought conditions using tree height could be possible.

### 4.5 Multi‐trait selection

The tested multi‐trait index selection scenarios showed that the integration of both wood density and a drought response component along with tree height resulted in the best improvement overall in the context of the drought episodes detected at both experimental sites. This scenario represented a selection for tree growth (height), wood quality (density), as well as the resistance or resilience components of drought response. While we found opposite phenotypic correlations between growth traits (DBH, tree height) and the resistance component of drought response between both sites, we found no opposite significant correlations at the genetic level. Even for the Watford study site, the resistance component of drought response had a significant positive genetic correlation with tree height. This pattern allowed selecting for height, usually the priority trait for improvement of white spruce, while only slightly affecting drought response components at the Normandin study site, or even improving the resistance to drought at Watford in single‐trait selection for height. The negative genetic correlations between wood density and both radial and height growth have been well documented for white spruce (Lenz et al., 2013) and they have also been observed in the present study. Therefore, the scenario S1 considering only tree height showed a reduction of wood density at the Normandin site and a very small increase at the Watford site. The integration of either the resistance or the resilience component of drought response along with tree height showed an improvement for drought response components, but no effect or only a small negative impact on the gain in wood density.

The best multi‐trait selection scenario was therefore to integrate either the resistance or the resilience component of drought response and wood density with tree height. The genetic gain estimated for wood density was still modest for scenarios S4 and S5, but quite in line with expectations for a weight of 0.6 on tree height, given its high priority in the objectives of the white spruce breeding program. All drought response traits tested had a positive gain with these scenarios. Thus, even when selecting for wood density as well as for the resistance or the resilience component of drought response and therefore, giving up a 0.4 weight in the selection index, tree height still had a significant genetic gain with scenarios S4 and S5. For example, S4, using the GBLUP method, resulted in 12.2% out of the maximum 12.9% genetic gain for tree height at the Normandin study site and 8.6% out of the maximum 10.0% at the Watford site. The only drought response trait that we could estimate at both sites was the resistance component, which was the least heritable of the four components of drought response based on evaluations for the Normandin study site, but with higher heritability at the Watford study site. While our scenario considering the resistance component of drought response showed an improvement of all traits at the Normandin site, the higher heritability and estimated genetic gain for the resilience component of drought response are indicative of a more efficient integration of drought response in selection schemes using this trait. However, we could not verify this assumption trend on both sites given the thinning of the Watford plantation during fall 2012 that precluded the estimation of the postdrought effects.

### 4.6 Cumulative impacts of drought episodes on growth productivity

One should remain critical when inspecting estimated genetic gains regarding the various components of drought response. While we estimated similar maximum genetic gain for drought response traits when compared to conventional traits, their long‐term impact appears to be quite different. Indeed, these drought response components as we calculated them using the method of Lloret et al. (2011), only implicated radial growth of 5 of the 18 total years that we considered using the cumulative rings in the DBH trait (or total tree height). Hence, the genetic gains estimated for drought response in this study might not be as important on cumulative long‐term radial growth as for selecting directly for DBH. It is likely that exceptional drought episodes as those documented by Depardieu et al. (2020) for white spruce and by Montwé et al. (2016) for lodgepole pine (*Pinus contorta*) might implicate increased genetic gains in cumulative growth when selecting for drought response traits. In comparison with our study where we found 6% and 12% mean annual growth loss on the Normandin and Watford experimental sites, respectively, due to the two detected drought episodes (see detrended growth values in Figure 1), a detrending of Depardieu et al. (2020) complete dataset, using the same detrending method as that used in our study, detected consecutive annual growth losses of 15% and 21% for the current and following year, respectively, after the severe drought episode they observed. This severe drought episode was also followed by another episode just 1 year after a partial recovery of the radial growth. These calculations indicate that even a moderate selection for drought response should increase long‐term radial growth in the context of increasing frequency and intensity of drought episodes whose effects are cumulative on radial growth. Drought episodes in close succession whose first recovery period is overlapped by a second growth slowdown have already been observed (Depardieu et al., 2020). The increasing frequency and intensity of drought episodes and the possibility of facing shorter delays between drought episodes than the recovery time (Schwalm et al., 2017) put into perspective the importance of studying growth response of temperate–boreal tree species to drought episodes before suffering important economic losses that could be partially avoided by planting more drought‐resilient trees.

## 5 CONCLUSIONS

With the constantly decreasing costs of genotyping and the shortening of breeding cycles times, GS represents a powerful tool regarding the rapid integration of adaptive traits to extreme climatic conditions very early in the tree breeding cycles (Bousquet et al., 2021; Park et al., 2016). While a single selection scenario permitted an improvement for all traits on both study sites, some contradicting phenotypic correlations found in this study raise the need to investigate the effects from different drought episodes on several sites and at different ages. Indeed, we observed that more vigorous growth during the tree lifespan corresponded to better resistance in conditions where the drought episode had more severe effects on current year growth. Thus, multi‐data analyses of physiological, genetic, and genomic nature under broad temporal and environmental scales are likely worthwhile options to better comprehend the quantitative genetics of the response to drought stress in tree species (Depardieu et al., 2021; Opgenoorth & Rellstab, 2021). This is especially feasible given that extensive genomic resources and common garden long‐term experiments are now accessible for many tree species, including spruces (Bousquet et al., 2021).

The increasing frequency and intensity of drought episodes due to climate change urge tree breeders to integrate drought response in tree breeding programs to prevent future reforested stock from important growth slowdown and economic loss. Relying on genetic variation in drought response from young white spruces originating from polycrosses implicating different provenances in eastern Canada, this study has highlighted possible paths to consider drought response in the multi‐trait genetic improvement of the widespread conifer white spruce using genomic selection.

## CONFLICT OF INTEREST

The authors declare that they have no conflict of interest.

## Supporting information

Fig S1‐S12

Table S1‐S8

## Data Availability

The SNP genotyping chip that was used has been made available by Lenz, Nadeau, Azaiez, et al. (2020). The SNP genotyping dataset is subject to another publication and will be made available to the public domain afterwards. The phenotypic datasets used in this study are the priority of the white spruce tree breeding program of the province of Quebec and Natural Resources Canada and will be available in institutional databases. Data will be shared upon request to the corresponding author and in agreement with institutional data sharing policies.
