# Supplementary material for: Breeding for adaptation to climate change: genomic selection for drought response in a white spruce multi‐site polycross test
Source: Evol Appl. 2022 Feb 28;15(3):383–402. doi: 10.1111/eva.13348 (PMC8965362; doi:10.1111/eva.13348)
Supplement: Supplementary file 1 — Fig S1‐S12 [file EVA-15-383-s001.docx]

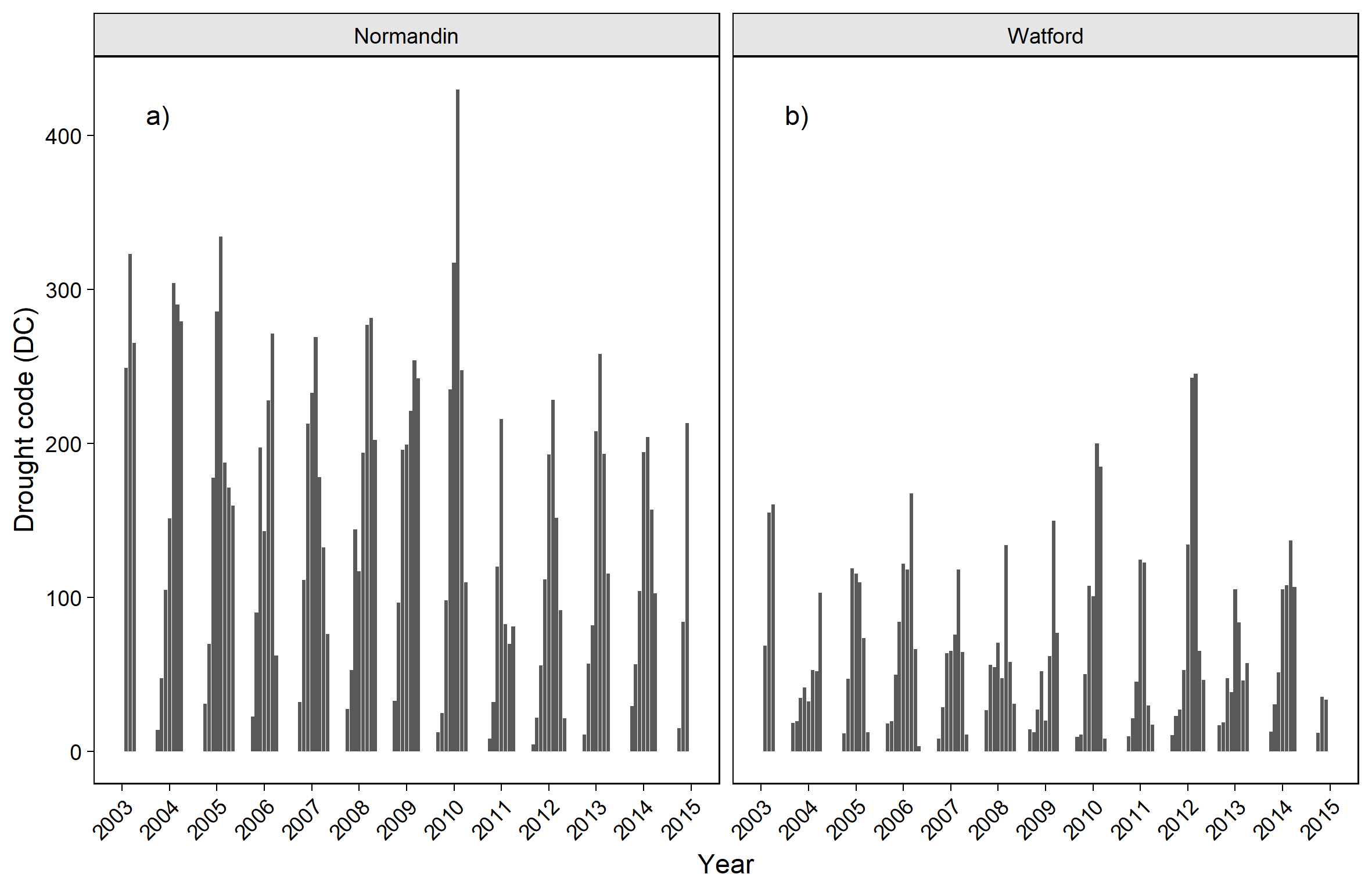


**Figure S1**. Monthly drought code for the period 2003-2015 for both Normandin (a) and Watford (b) study sites. The position of the year on the x-axis corresponds to the separation between the months of June and July.


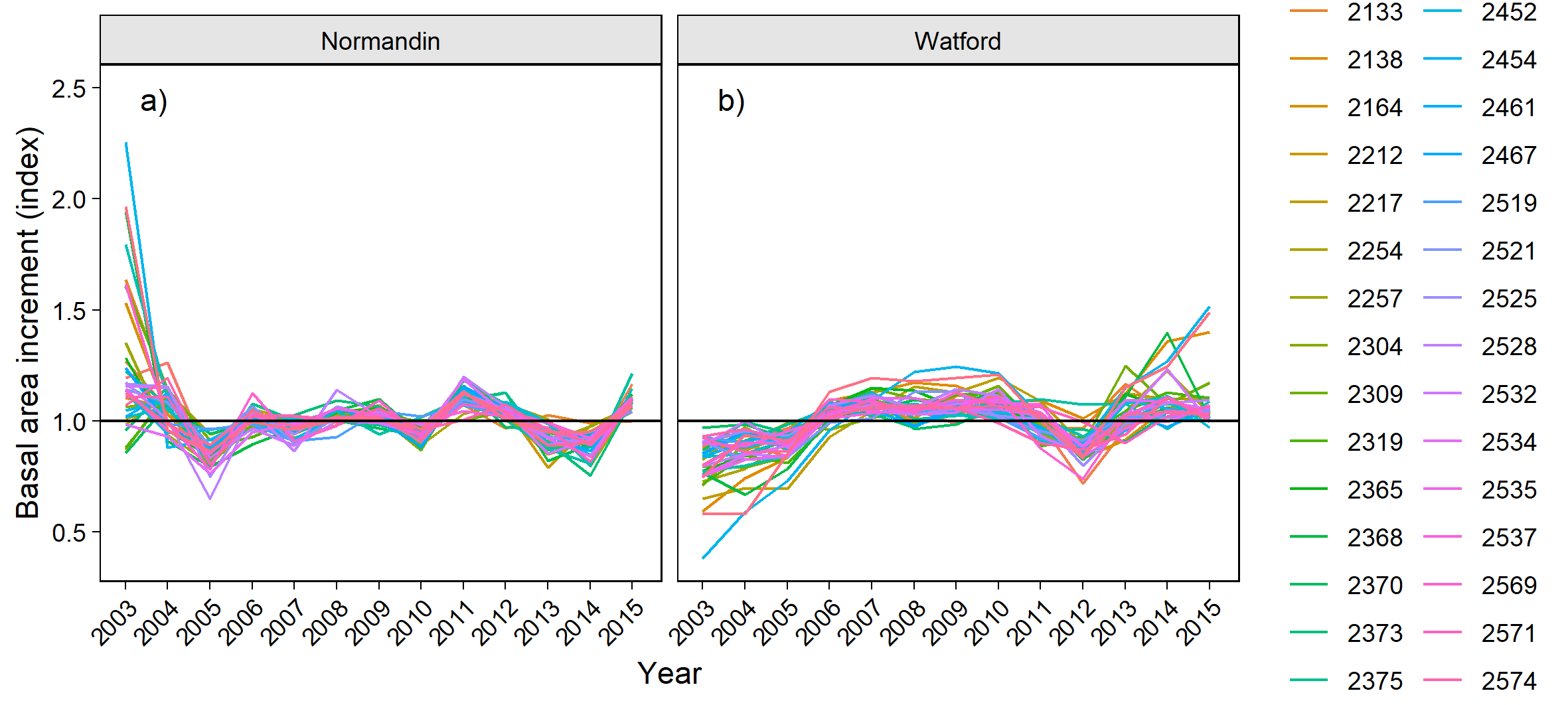


**Figure S2**. Mean detrended BAI (index) for each white spruce family at the Normandin (a) and Watford (b) study sites. Mean chronologies were generated using the dplR package as described in the Material and Methods. These chronologies were used for the estimation of correlations between radial growth and monthly drought code (DC) at the family level.


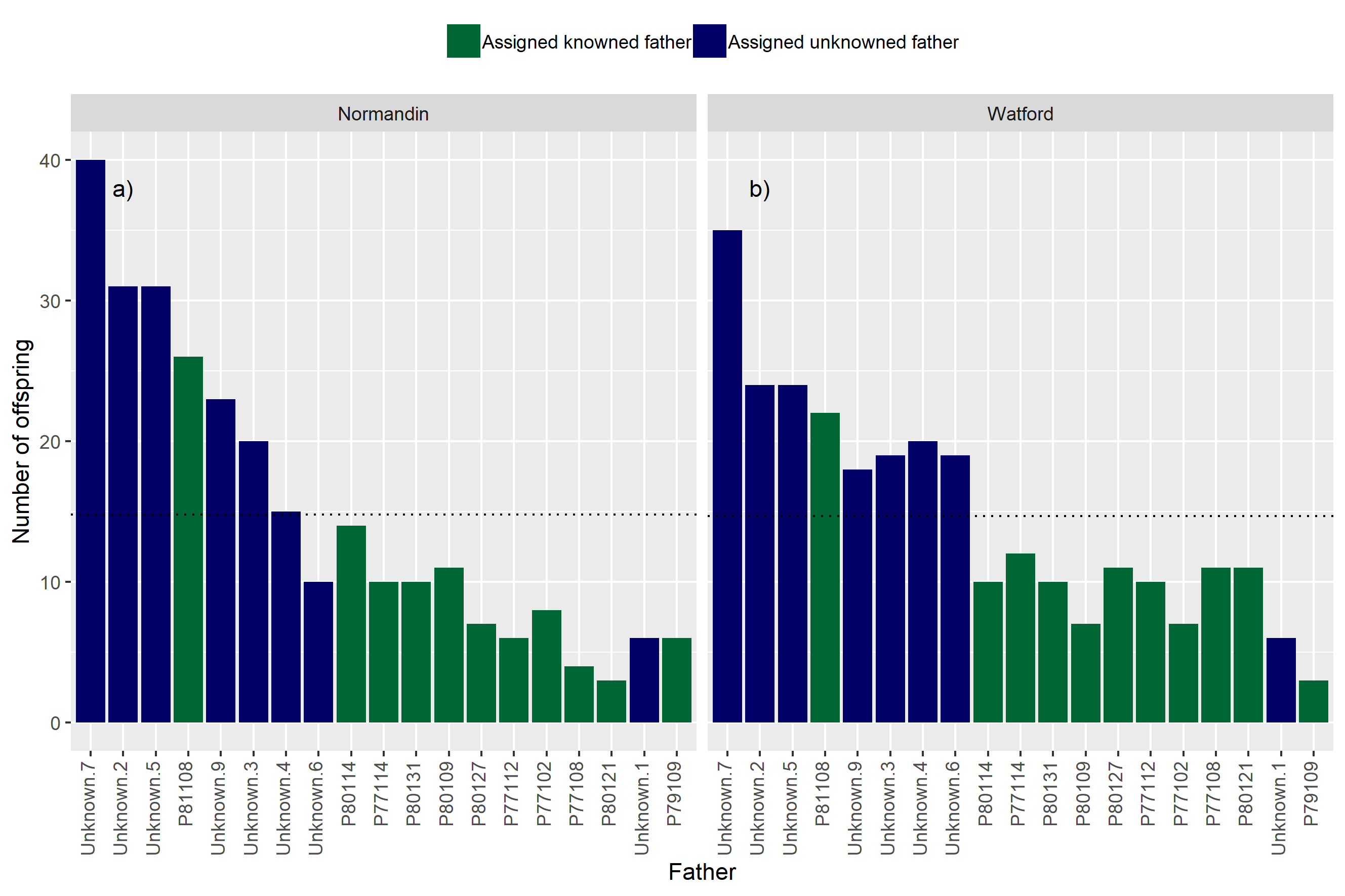


**Figure S3**. Number of white spruce offspring per non genotyped pollen donor for both Normandin (a) and Watford (b) study sites. In blue, unknown fathers, in green known fathers. The dashed line represents the expected number of offspring per pollen donor with equal reproductive success of 14.8 and 14.7 offspring per father at the Normandin and Watford study sites, respectively. For the paternal recovery methodology, please see Lenz et al. (2020).


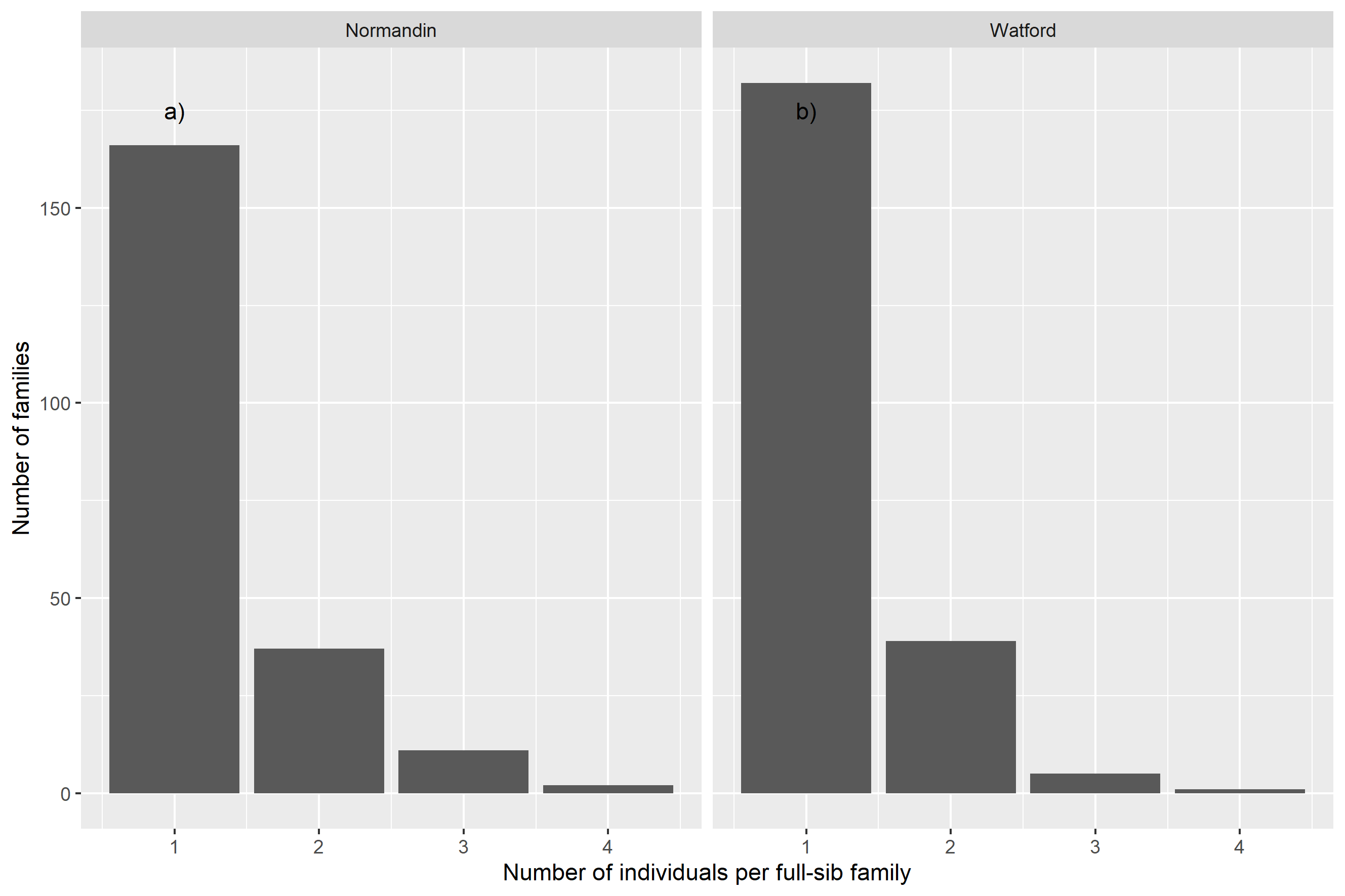


**Figure S4**. Histogram of full-sub family size recovered from the polycross families at the Normandin (a) and Watford (b) study sites.


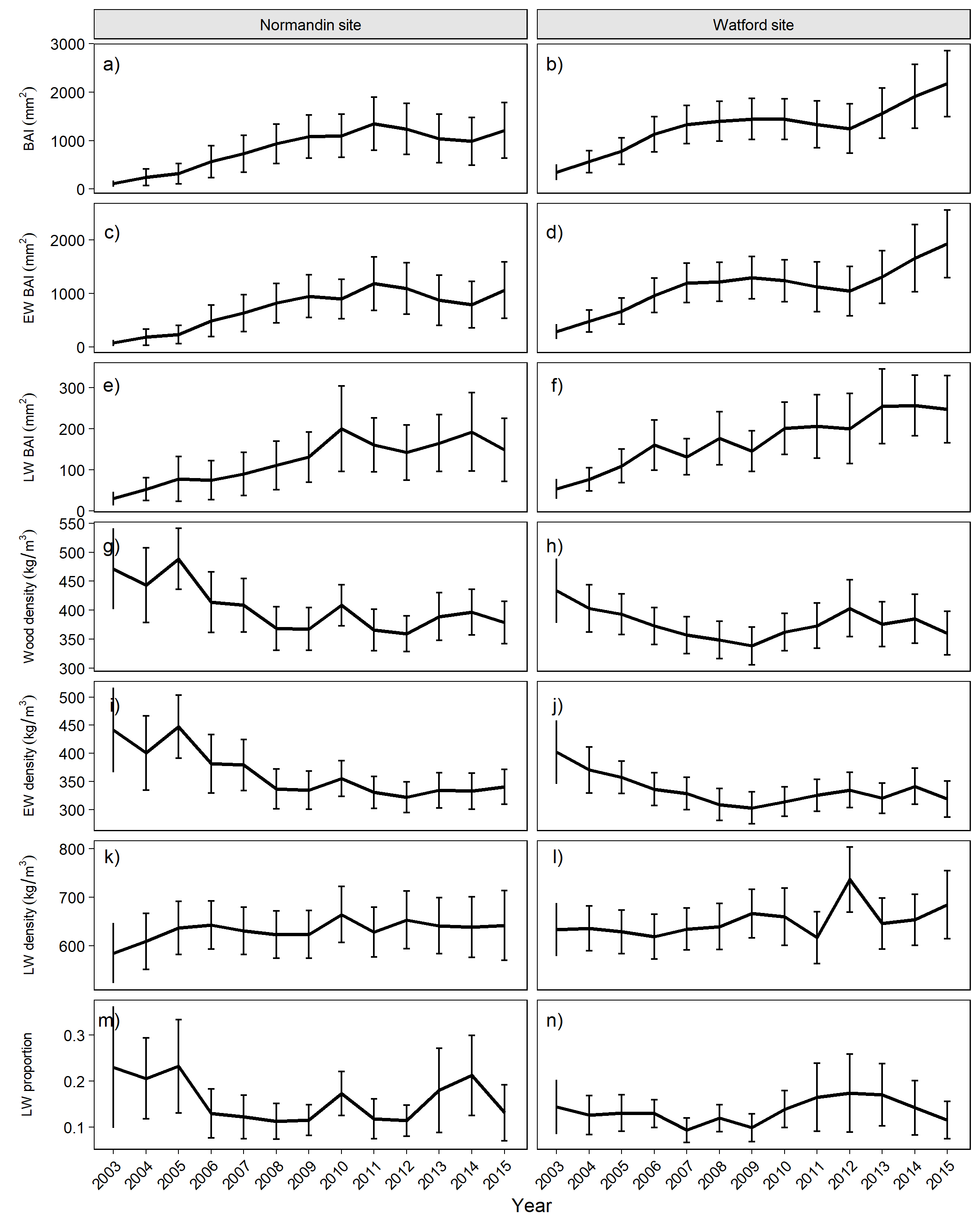


**Figure S5**. Annual mean and standard deviation for cumulative and earlywood (EW) and latewood (LW) components of basal area increment (BAI) and wood density for both Normandin and Watford study sites.


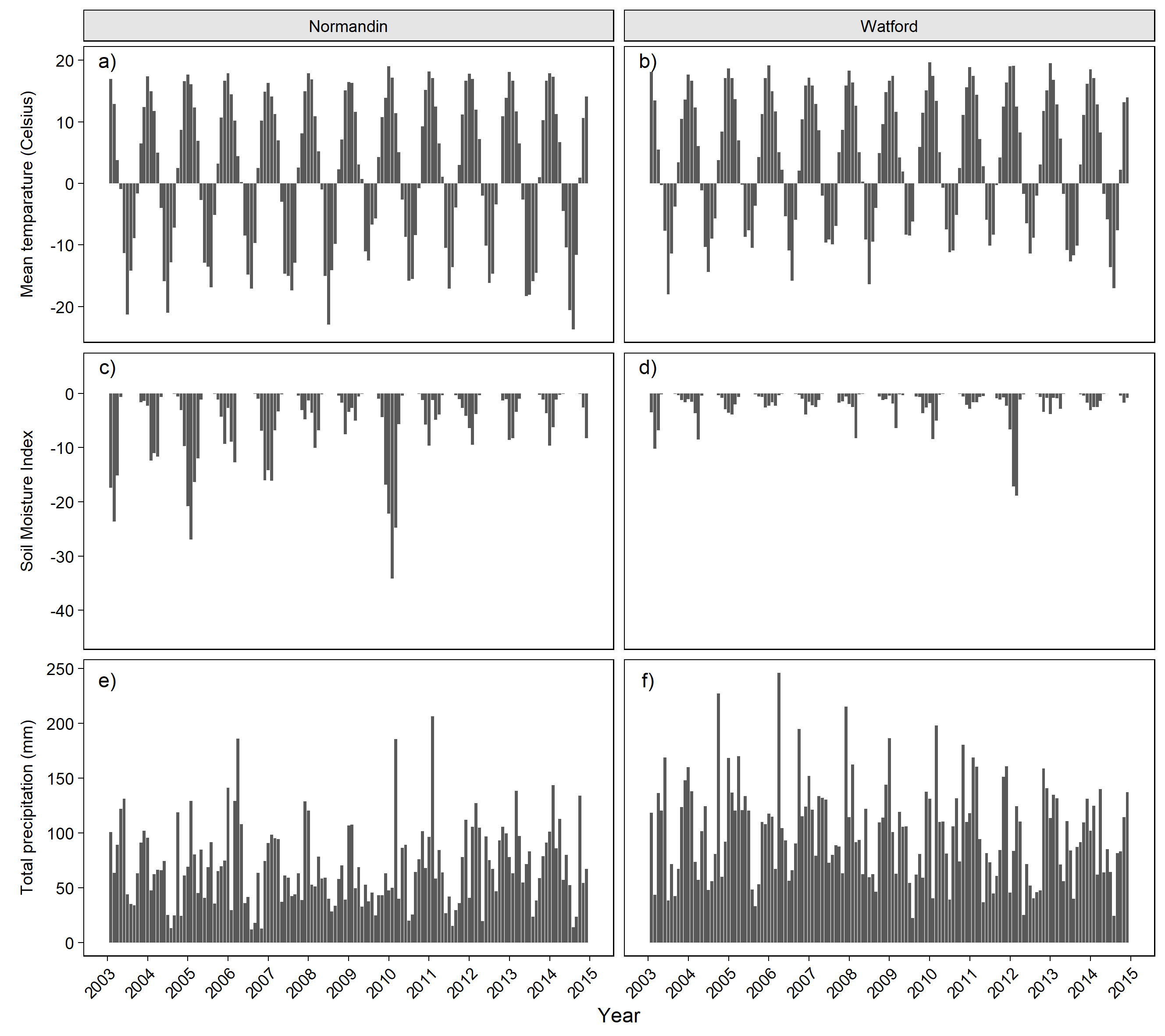


**Figure S6**. Raw monthly values of mean temperature (a, b), soil moisture index (SMI) (c, d) and total precipitation (e, f) at both Normandin and Watford study sites. The position of the year on the x-axis corresponds to the separation between the months of June and July. Monthly soil moisture index (SMI) has been subtracted by 100 for visualisation purposes. Higher values of SMI (near 0 on the figure) correspond to humid conditions.


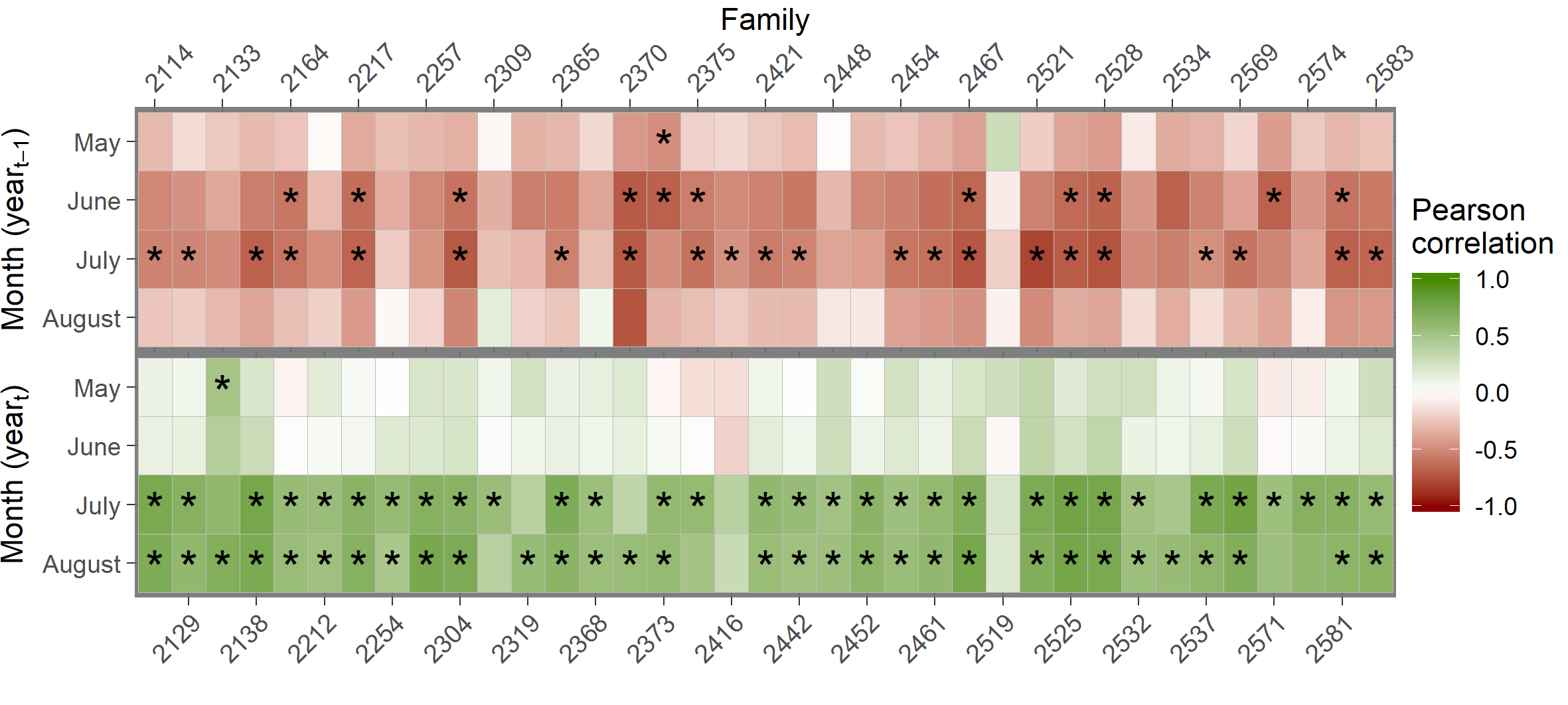


**Figure S7**. Pearson correlations between mean family basal area increment (BAI) indices and monthly soil moisture index (SMI) for the Normandin study site. Families are presented on x-axis, months on y-axis. The preceding year months appear on the upper half, and the current year months, on the lower half. Significant correlations (*P* < 0.05) as calculated with the “dcc” function of the treeclim R package are shown by an asterisk.


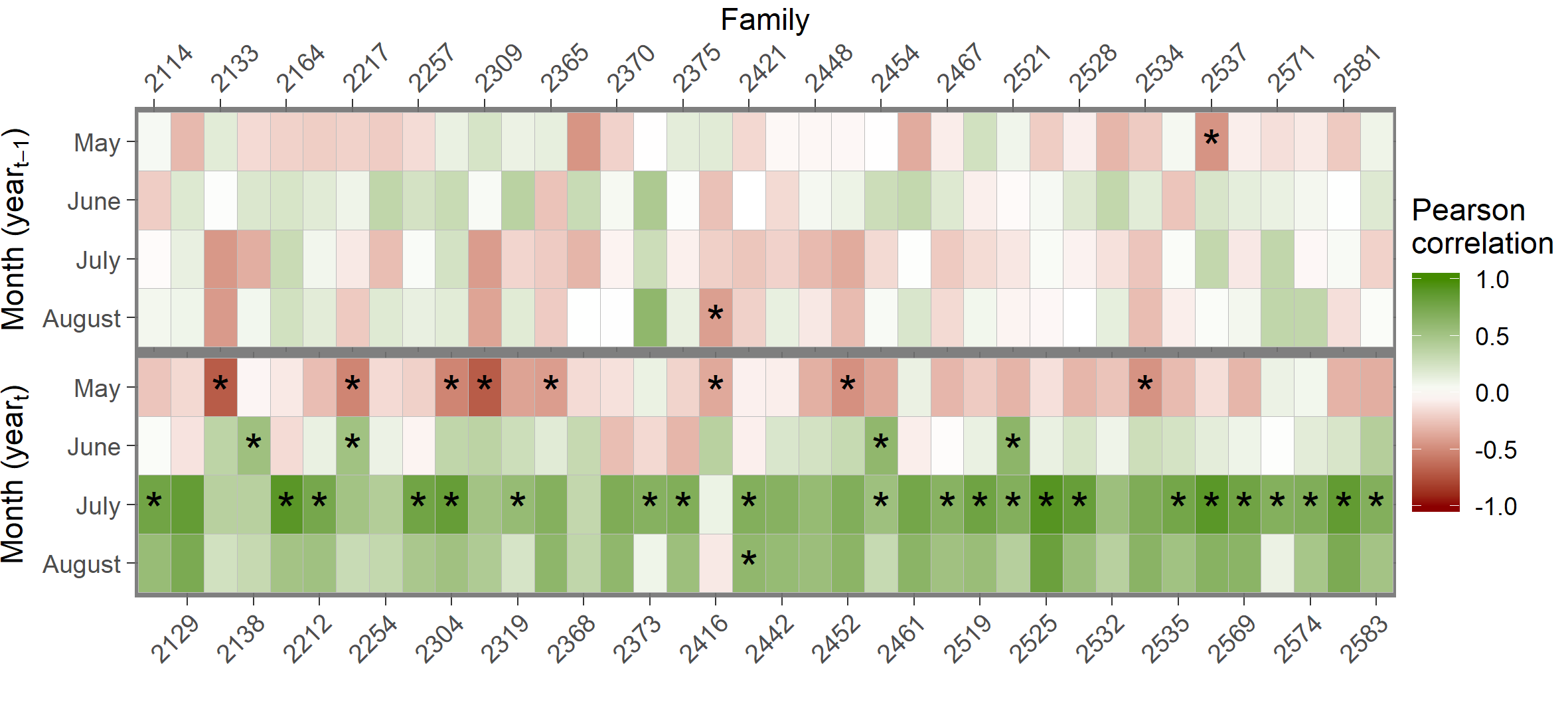


**Figure S8**. Pearson correlations between mean family basal area increment (BAI) indices and monthly soil moisture index (SMI) for the Watford study site. High values of SMI reflect humid conditions. Families are presented on x-axis, months on y-axis. The preceding year months appear on the upper half, and the current year months, on the lower half. Significant correlations (*P* < 0.05) as calculated with the “dcc” function of the treeclim R package are shown by an asterisk.


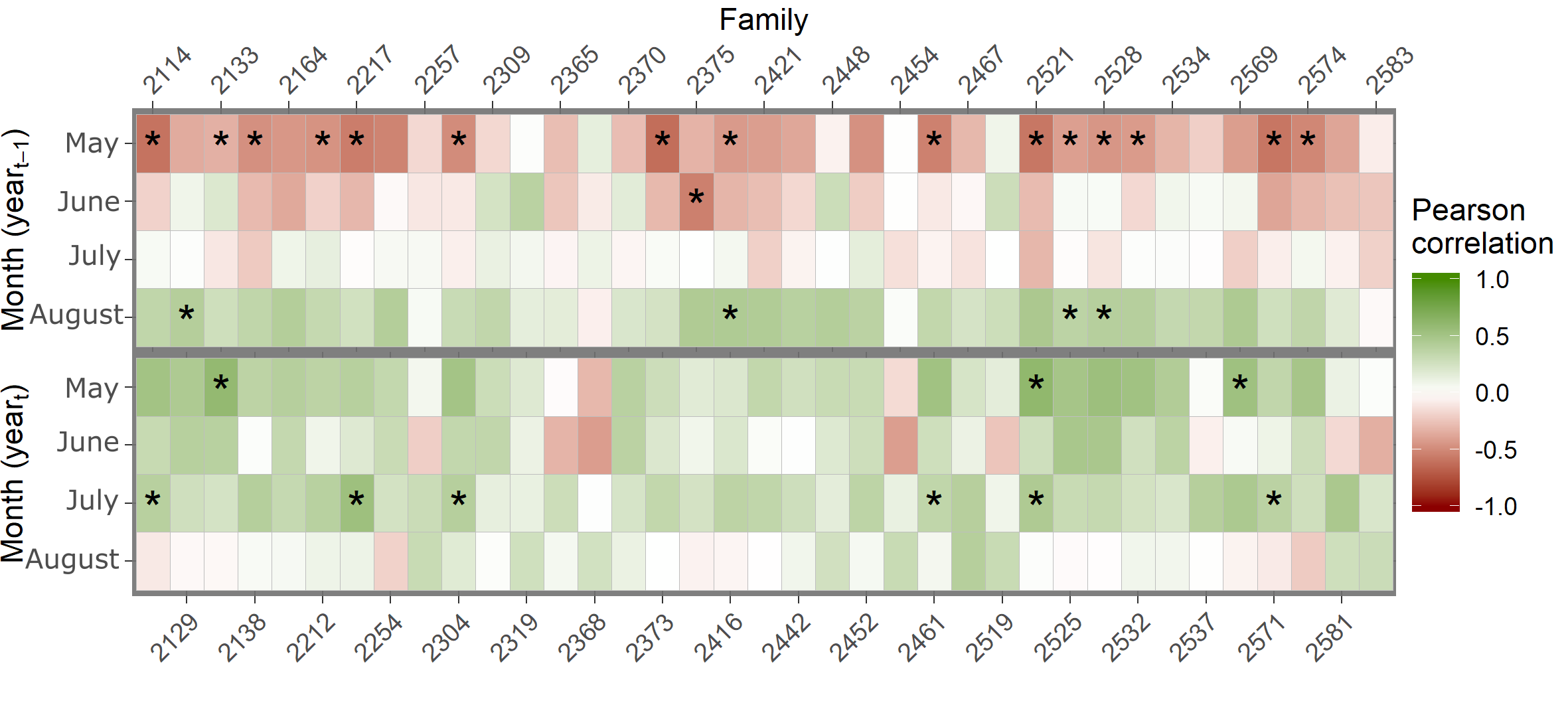


**Figure S9**. Pearson correlations between mean family basal area increment (BAI) indices and monthly total precipitation for the Normandin study site. Families are presented on x-axis, months on y-axis. The preceding year months appear on the upper half, and the current year months, on the lower half. Significant correlations (*P* < 0.05) as calculated with the “dcc” function of the treeclim R package are shown by an asterisk.


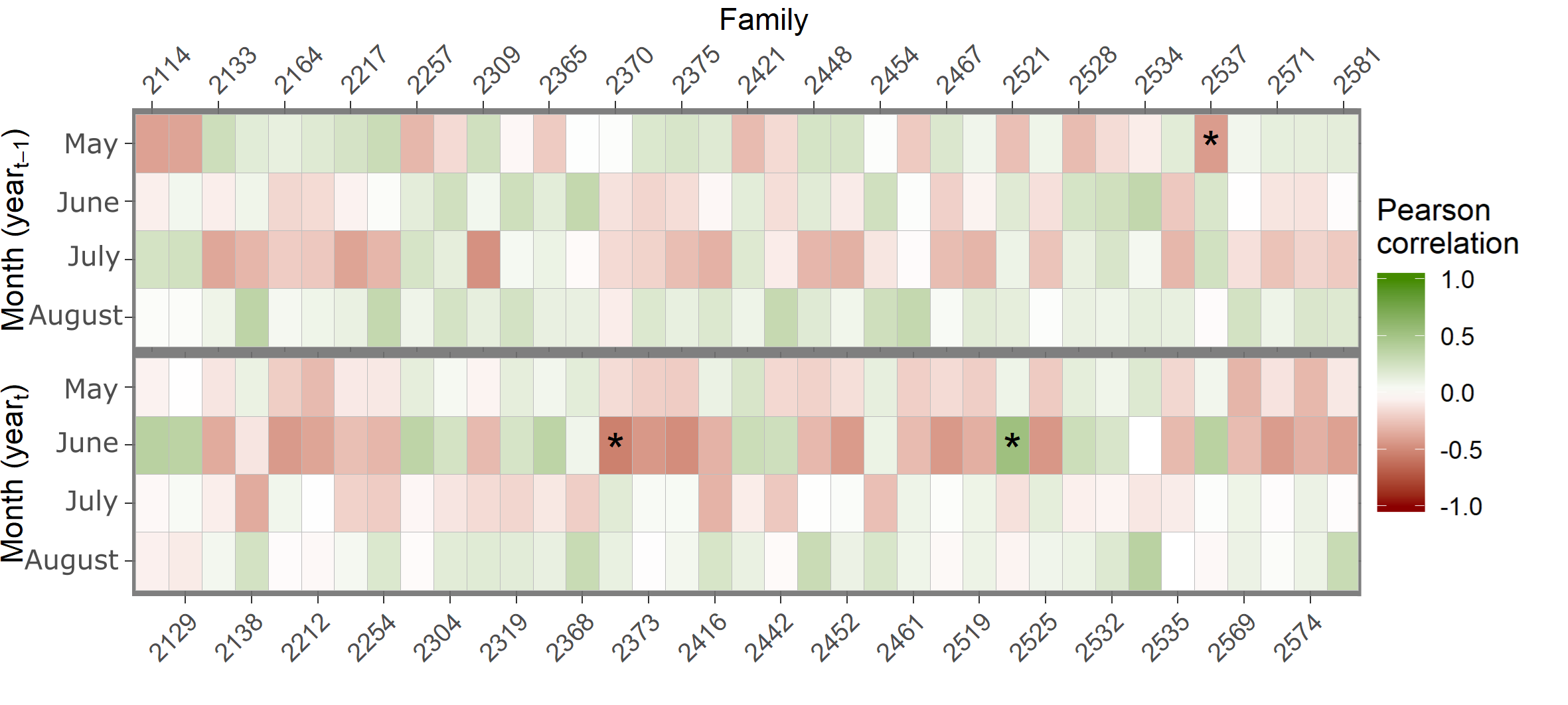


**Figure S10**. Pearson correlations between mean family basal area increment (BAI) indices and monthly total precipitation for the Watford study site. Families are presented on x-axis, months on y-axis. The preceding year months appear on the upper half, and the current year months, on the lower half. Significant correlations (*P* < 0.05) as calculated with the “dcc” function of the treeclim R package are shown by an asterisk.


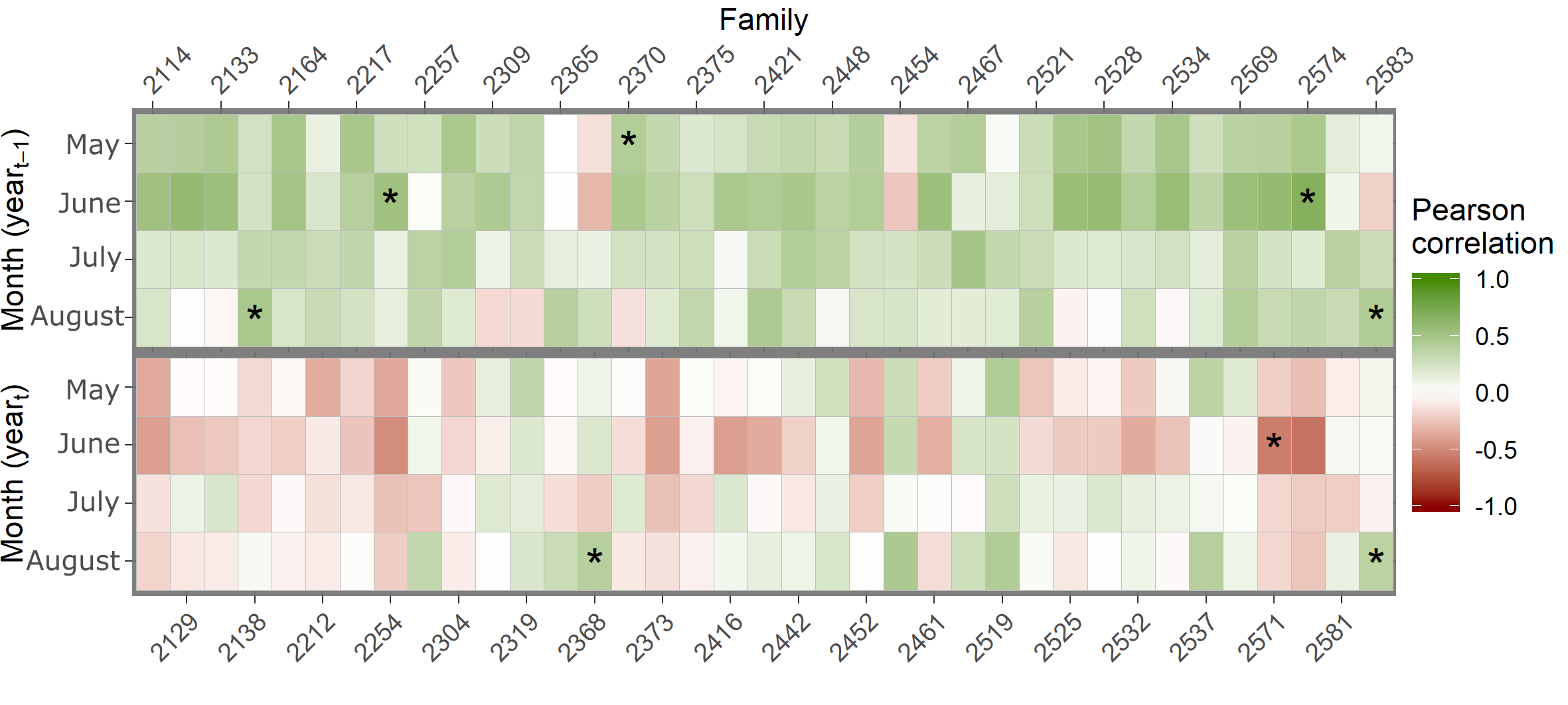


**Figure S11**. Pearson correlations between mean family basal area increment (BAI) indices and monthly mean temperature for the Normandin study site. Families are presented on x-axis, months on y-axis. The preceding year months appear on the upper half, and the current year months, on the lower half. Significant correlations (*P* < 0.05) as calculated with the “dcc” function of the treeclim R package are shown by an asterisk.


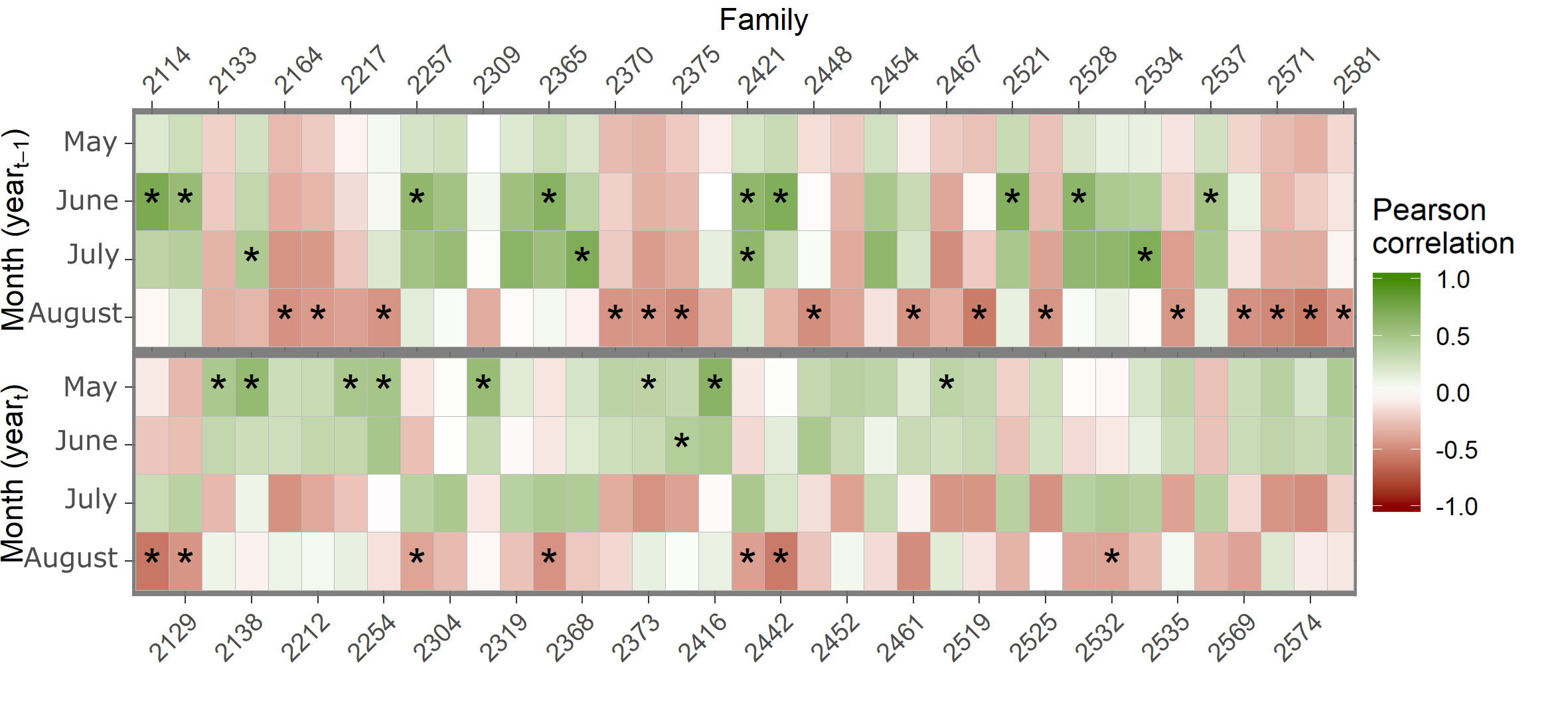


**Figure S12**. Pearson correlations between mean family basal area increment (BAI) indices and monthly mean temperature for the Watford study site. Families are presented on x-axis, months on y-axis. The preceding year months appear on the upper half, and the current year months, on the lower half. Significant correlations (*P* < 0.05) as calculated with the “dcc” function of the treeclim R package are shown by an asterisk.

**References to supplementary figures**

Lenz, P. R. N., Nadeau, S., Azaiez, A., Gérardi, S., Deslauriers, M., Perron, M., Isabel, N., Beaulieu, J., and Bousquet, J. (2020). Genomic prediction for hastening and improving efficiency of forward selection in conifer polycross mating designs: an example from white spruce. *Heredity, 124*, 562-578. <https://doi.org/10.1038/s41437-019-0290-3>
