# Supplementary material for: Breeding for adaptation to climate change: genomic selection for drought response in a white spruce multi‐site polycross test
Source: Evol Appl. 2022 Feb 28;15(3):383–402. doi: 10.1111/eva.13348 (PMC8965362; doi:10.1111/eva.13348)
Supplement: Supplementary file 2 — Table S1‐S8 [file EVA-15-383-s002.docx]

**Table S1**. Mean and standard deviation values for both raw and detrended basal area increment (BAI) values at the Normandin and Watford study sites. The mean chronologies were calculated using the “chron” function of the dplR R package.

|  | Normandin site | |  | Watford site | |
| --- | --- | --- | --- | --- | --- |
| Years | Raw BAI | Detrended BAI |  | Raw BAI | Detrended BAI |
| 2003 | 101.5 (64.5) | 1.15 (4.65) |  | 340.7 (162.5) | 0.83 (0.55) |
| 2004 | 220.9 (172.6) | 1.07 (1.12) |  | 560.4 (227.4) | 0.89 (0.24) |
| 2005 | 298.6 (210.7) | 0.84 (0.37) |  | 774.2 (273.5) | 0.89 (0.15) |
| 2006 | 542.8 (332.0) | 1.02 (0.20) |  | 1116.7 (368.7) | 1.04 (0.11) |
| 2007 | 711.1 (381.1) | 0.96 (0.15) |  | 1320.6 (396.4) | 1.07 (0.13) |
| 2008 | 920.3 (408.2) | 1.02 (0.12) |  | 1386.5 (411.1) | 1.05 (0.16) |
| 2009 | 1066.3 (445.8) | 1.02 (0.14) |  | 1437.7 (426.3) | 1.07 (0.15) |
| 2010 | 1075.0 (446.8) | 0.94 (0.16) |  | 1430.1 (423.9) | 1.08 (0.14) |
| 2011 | 1319.4 (550.5) | 1.11 (0.19) |  | 1324.8 (487.9) | 0.99 (0.15) |
| 2012 | 1213.4 (526.9) | 1.04 (0.15) |  | 1235.0 (509.2) | 0.88 (0.18) |
| 2013 | 1022.3 (500.5) | 0.93 (0.20) |  | 1547.9 (519.9) | 1.03 (0.23) |
| 2014 | 965.6 (495.4) | 0.90 (0.17) |  | 1904.9 (660.6) | 1.07 (0.32) |
| 2015 | 1191.7 (576.1) | 1.08 (0.16) |  | 2179.8 (680.7) | 1.03 (0.43) |

**Table S2**. Pearson correlation coefficients between mean site basal area increment (BAI) indices and monthly drought code (DC) of current year at the Normandin and Watford study sites. Only months showing a significant correlation for at least one site are presented. A confidence interval defined by a p-value < 0.05 as calculated with the “dcc” function of the treeclim R package is shown. Significant correlations (*P* < 0.05) are shown in bold font.

|  | Normandin site | |  | Watford site | |
| --- | --- | --- | --- | --- | --- |
| Month | Correlation | Confidence interval |  | Correlation | Confidence interval |
| July | **-0.55** | **[ -0.84 , -0.03 ]** |  | **-0.62** | **[ -0.81 , -0.36 ]** |
| August | **-0.73** | **[ -0.95 , -0.29 ]** |  | -0.49 | [ -0.93 , 0.30 ] |

**Table S3**. Genetic (above the diagonal) and phenotypic (below the diagonal) correlations between all traits^a^ studied for the Normandin study site using GBLUP^b^. Standard errors are shown in parentheses along with significance level indicated by asterisks^c^.

| Trait type | Drought response | | | | Growth | | Wood quality | | | | Growth | |
| --- | --- | --- | --- | --- | --- | --- | --- | --- | --- | --- | --- | --- |
| Traits | Recovery | Relative resilience | Resilience | Resistance | Height | DBH | Acoustic velocity | Wood density | EW density | LW  density | EW  area | LW  area |
| Recovery | - | 0.99† | 0.79** (0.15) | -0.06 (0.39) | 0.41 (0.24) | 0.22 (0.27) | -0.18 (0.29) | -0.32 (0.27) | -0.23 (0.27) | 0.36 (0.39) | 0.20 (0.29) | -0.12 (0.31) |
| Relative resilience | 0.97†  (0.00) | - | 0.85** (0.11) | 0.06 (0.39) | 0.44 (0.24) | 0.18 (0.27) | -0.15 (0.28) | -0.37 (0.25) | -0.28 (0.26) | 0.29 (0.39) | 0.16 (0.30) | -0.17 (0.31) |
| Resilience | 0.72*** (0.03) | 0.81*** (0.02) | - | 0.58 (0.26) | 0.35 (0.26) | -0.14 (0.30) | -0.09 (0.30) | -0.42 (0.26) | -0.31 (0.26) | -0.05 (0.40) | -0.13 (0.32) | -0.55* (0.28) |
| Resistance | -0.05 (0.06) | 0.06 (0.06) | 0.64*** (0.04) | - | 0.04 (0.33) | -0.49 (0.27) | 0.07 (0.33) | -0.31 (0.32) | -0.21 (0.31) | -0.54 (0.40) | -0.47 (0.30) | -0.66** (0.23) |
| Height | 0.19** (0.06) | 0.16** (0.06) | 0.04 (0.07) | -0.15** (0.06) | - | 0.65** (0.13) | 0.07 (0.23) | 0.07 (0.24) | 0.08 (0.23) | 0.16 (0.34) | 0.65** (0.15) | 0.63** (0.15) |
| DBH | 0.18** (0.06) | 0.13* (0.06) | -0.08 (0.06) | -0.31*** (0.06) | 0.63*** (0.04) | - | -0.10 (0.23) | -0.38 (0.20) | -0.35 (0.21) | -0.15 (0.33) | † | 0.96*** (0.03) |
| Acoustic velocity | -0.02 (0.06) | -0.04 (0.06) | -0.03 (0.06) | 0.00 (0.06) | 0.18** (0.07) | -0.11 (0.07) | - | 0.34 (0.21) | 0.30 (0.21) | 0.42 (0.31) | -0.11 (0.25) | -0.01 (0.24) |
| Wood density | -0.17** (0.06) | -0.15* (0.06) | -0.12 (0.06) | 0.00 (0.06) | -0.17** (0.07) | -0.37*** (0.06) | 0.17** (0.07) | - | † | 0.58 (0.21) | -0.38 (0.22) | -0.05 (0.24) |
| EW density | -0.13 (0.06) | -0.11 (0.06) | -0.09 (0.06) | -0.01 (0.06) | -0.15** (0.07) | -0.31*** (0.06) | 0.13 (0.07) | † | - | 0.40 (0.26) | -0.34 (0.22) | -0.09 (0.24) |
| LW density | 0.02 (0.06) | 0.02 (0.06) | 0.01 (0.06) | -0.02 (0.06) | 0.02 (0.06) | -0.15** (0.06) | 0.24*** (0.06) | 0.95*** (0.12) | 0.54*** (0.05) | - | -0.14 (0.35) | 0.13 (0.37) |
| EW area | 0.16** (0.06) | 0.12 (0.06) | -0.04 (0.06) | -0.22** (0.06) | 0.61*** (0.04) | † | -0.08 (0.07) | -0.42*** (0.06) | -0.36*** (0.06) | -0.16** (0.06) | - | 0.93*** (0.04) |
| LW area | 0.05 (0.06) | 0.01 (0.07) | -0.13 (0.06) | -0.24** (0.06) | 0.57*** (0.05) | 0.86*** (0.02) | -0.05 (0.07) | -0.18** (0.07) | -0.18** (0.07) | -0.20*** (0.06) | 0.87*** (0.02) | - |

^a^ See Table 1 for a full description of traits.
^b^ The model fitted for each combination of traits is described in Equation 5.
^c^ Levels of statistical significance: * *P* < 0.05 | ** *P* < 0.01 | *** *P* < 0.001 | † convergence failed.

**Table S4**. Genetic (above the diagonal) and phenotypic (below the diagonal) correlations between all traits^a^ studied for the Watford study site using GBLUP^b^. Standard errors are shown in parentheses along with significance level indicated by asterisks^c^.

| Trait types | Drought response | Growth | | Wood quality | | | | Growth | |
| --- | --- | --- | --- | --- | --- | --- | --- | --- | --- |
| Traits | Resistance | Height | DBH | Acoustic velocity | Wood density | EW density | LW density | EW area | LW area |
| Resistance | - | 0.59** (0.19) | 0.45 (0.25) | 0.05 (0.26) | 0.18 (0.27) | 0.24 (0.27) | 0.13 (0.29) | 0.40 (0.28) | 0.34 (0.27) |
| Height | 0.49*** (0.05) | - | 0.83*** (0.09) | 0.31 (0.19) | -0.33 (0.21) | -0.27 (0.22) | 0.10 (0.23) | 0.86*** (0.10) | 0.47** (0.19) |
| DBH | 0.49*** (0.05) | 0.67*** (0.04) | - | -0.06 (0.23) | -0.65** (0.17) | -0.67** (0.17) | -0.39 (0.24) | 0.99*** (0.03) | 0.70** (0.14) |
| Acoustic velocity | 0.12* (0.07) | 0.19** (0.07) | -0.11 (0.07) | - | 0.31 (0.19) | 0.30 (0.19) | 0.55** (0.16) | -0.01 (0.25) | -0.05 (0.22) |
| Wood density | -0.05 (0.07) | -0.09 (0.07) | -0.37*** (0.06) | 0.28*** (0.06) | - | 0.95*** (0.02) | 0.87*** (0.08) | -0.71** (0.17) | -0.31 (0.23) |
| EW density | -0.03 (0.07) | -0.05 (0.07) | -0.31*** (0.06) | 0.26*** (0.06) | 1.05*** (0.04) | - | 0.76** (0.11) | -0.75** (0.16) | -0.50** (0.21) |
| LW density | 0.10 (0.07) | 0.16** (0.07) | -0.12 (0.07) | 0.40*** (0.06) | 0.85*** (0.06) | 0.64*** (0.04) | - | -0.48* (0.24) | 0.09 (0.27) |
| EW area | 0.41*** (0.05) | 0.59*** (0.04) | 0.90*** (0.01) | -0.11 (0.07) | -0.41*** (0.05) | -0.35*** (0.06) | -0.10 (0.07) | - | 0.77** (0.12) |
| LW area | 0.33*** (0.06) | 0.43*** (0.05) | 0.66*** (0.04) | -0.07 (0.07) | 0.01 (0.07) | -0.06* (0.07) | -0.07 (0.07) | 0.69*** (0.03) | - |

^a^ See Table 3.1 for a full description of traits.
^b^ The model fitted for each combination of traits is described in Equation 5.
^c^ Levels of statistical significance: * *P* < 0.05 | ** *P* < 0.01 | *** *P* < 0.001 | † convergence failed.

**Table S5**. Genetic (above the diagonal) and phenotypic (below the diagonal) correlations between all traits^a^ studied for the Normandin study site Normandin using ABLUP^b^. Standard errors are shown in parentheses along with significance level indicated by asterisks^c^.

| Trait type | Drought response | | | | Growth | | Wood quality | | | | Growth | |
| --- | --- | --- | --- | --- | --- | --- | --- | --- | --- | --- | --- | --- |
| Traits | Recovery | Relative resilience | Resilience | Resistance | Height | DBH | Acoustic velocity | Wood density | EW density | LW  density | EW  area | LW  area |
| Recovery | - | 0.99† | 0.84** (0.12) | 0.00 (0.41) | 0.40 (0.25) | 0.16 (0.28) | -0.15 (0.29) | -0.26 (0.27) | -0.20 (0.27) | 0.49 (0.41) | 0.15 (0.30) | -0.23 (0.32) |
| Relative resilience | 0.97† (0.00) | - | 0.90** (0.08) | 0.12 (0.40) | 0.42 (0.25) | 0.11 (0.29) | -0.13 (0.28) | -0.27 (0.27) | -0.20 (0.27) | 0.46 (0.43) | 0.11 (0.30) | -0.29 (0.32) |
| Resilience | 0.72*** (0.03) | 0.81*** (0.02) | - | 0.55 (0.29) | 0.33 (0.29) | -0.08 (0.31) | -0.14 (0.30) | -0.36 (0.27) | -0.26 (0.27) | 0.08 (0.46) | -0.09 (0.33) | -0.54 (0.31) |
| Resistance | -0.04 (0.06) | 0.07 (0.06) | 0.64*** (0.04) | - | -0.07 (0.39) | -0.39 (0.34) | -0.03 (0.37) | -0.30 (0.35) | -0.20 (0.35) | -0.56 (0.42) | -0.40 (0.37) | -0.53 (0.33) |
| Height | 0.20** (0.06) | 0.16** (0.07) | 0.03 (0.07) | -0.16** (0.06) | - | 0.61** (0.17) | 0.28 (0.24) | -0.17 (0.26) | -0.11 (0.25) | 0.15 (0.41) | 0.57** (0.19) | 0.49* (0.21) |
| DBH | 0.18** (0.06) | 0.13* (0.07) | -0.08 (0.07) | -0.31*** (0.06) | 0.62*** (0.04) | - | -0.07 (0.26) | -0.48* (0.21) | -0.41 (0.21) | -0.03 (0.40) | † | 0.96*** (0.04) |
| Acoustic velocity | -0.02 (0.07) | -0.04 (0.07) | -0.03 (0.07) | 0.00 (0.06) | 0.21** (0.07) | -0.11 (0.07) | - | 0.23 (0.25) | 0.15 (0.24) | 0.36 (0.36) | -0.15 (0.27) | -0.02 (0.27) |
| Wood density | -0.17** (0.06) | -0.15* (0.07) | -0.12 (0.07) | -0.01 (0.06) | -0.19** (0.07) | -0.38*** (0.06) | 0.16** (0.07) | - | † | 0.48 (0.29) | -0.49* (0.21) | -0.19 (0.27) |
| EW density | -0.13 (0.07) | -0.12 (0.07) | -0.10 (0.07) | -0.02 (0.06) | -0.17** (0.07) | -0.31*** (0.06) | 0.12 (0.07) | † | - | 0.27 (0.35) | -0.41 (0.22) | -0.18 (0.26) |
| LW density | 0.03 (0.06) | 0.03 (0.06) | 0.01 (0.06) | -0.03 (0.06) | 0.02 (0.06) | -0.14** (0.06) | 0.24*** (0.06) | 1.00*** (0.14) | 0.53*** (0.05) | - | -0.11 (0.42) | 0.20 (0.45) |
| EW area | 0.16** (0.06) | 0.12 (0.06) | -0.04 (0.06) | -0.22** (0.06) | 0.60*** (0.04) | † | -0.08 (0.07) | -0.43*** (0.06) | -0.36*** (0.06) | -0.15** (0.06) | - | 0.93** (0.05) |
| LW area | 0.05 (0.06) | 0.01 (0.06) | -0.13 (0.06) | -0.22** (0.06) | 0.55*** (0.05) | 0.85*** (0.02) | -0.05 (0.07) | -0.19** (0.07) | -0.19** (0.07) | -0.20*** (0.06) | 0.87*** (0.02) | - |

^a^ See Table 1 for a full description of traits.
^b^ The model fitted for each combination of traits is described in Equation 5.
^c^ Levels of statistical significance: * *P* < 0.05 | ** *P* < 0.01 | *** *P* < 0.001 | † convergence failed.

**Table S6**. Genetic (above the diagonal) and phenotypic (below the diagonal) correlations between all traits^a^ studied for the Watford study site using ABLUP^b^. Standard errors are shown in parentheses along with significance level indicated by asterisks^c^.

| Trait types | Drought response | Growth | | Wood quality | | | | Growth | |
| --- | --- | --- | --- | --- | --- | --- | --- | --- | --- |
| Traits | Resistance | Height | DBH | Acoustic velocity | Wood density | EW density | LW density | EW area | LW area |
| Resistance | - | 0.50* (0.23) | 0.35 (0.30) | -0.07 (0.29) | 0.00 (0.31) | 0.08 (0.31) | 0.02 (0.35) | 0.32 (0.32) | 0.15 (0.33) |
| Height | 0.49*** (0.05) | - | 0.82** (0.11) | 0.26 (0.22) | -0.35 (0.24) | -0.29 (0.24) | -0.02 (0.28) | 0.86*** (0.12) | 0.47* (0.21) |
| DBH | 0.48*** (0.05) | 0.67*** (0.04) | - | -0.08 (0.26) | -0.68** (0.18) | -0.71** (0.18) | -0.42 (0.27) | 1.00** (0.03) | 0.69** (0.17) |
| Acoustic velocity | 0.11* (0.07) | 0.19** (0.07) | -0.11 (0.07) | - | 0.23 (0.22) | 0.19 (0.22) | 0.57** (0.18) | -0.01 (0.28) | -0.05 (0.26) |
| Wood density | -0.06 (0.07) | -0.09 (0.07) | -0.37*** (0.06) | 0.28*** (0.07) | - | 0.96*** (0.02) | 0.85*** (0.10) | -0.77** (0.17) | -0.21 (0.27) |
| EW density | -0.03 (0.07) | -0.05 (0.07) | -0.31*** (0.06) | 0.25*** (0.07) | 1.04*** (0.04) | - | 0.67** (0.16) | -0.83** (0.16) | -0.44 (0.25) |
| LW density | 0.09 (0.06) | 0.16** (0.07) | -0.12 (0.07) | 0.41*** (0.06) | 0.86*** (0.07) | 0.63*** (0.04) | - | -0.46 (0.29) | 0.28 (0.32) |
| EW area | 0.41*** (0.05) | 0.59*** (0.04) | 0.90*** (0.01) | -0.11 (0.07) | -0.42*** (0.05) | -0.36*** (0.06) | -0.09 (0.06) | - | 0.72** (0.16) |
| LW area | 0.32*** (0.06) | 0.43*** (0.05) | 0.66*** (0.04) | -0.08 (0.07) | 0.02 (0.07) | -0.05 (0.07) | -0.06 (0.07) | 0.69*** (0.03) | - |

^a^ See Table 1 for a full description of traits.
^b^ The model fitted for each combination of traits is described in Equation 5.
^c^ Levels of statistical significance: * *P* < 0.05 | ** *P* < 0.01 | *** *P* < 0.001 | † convergence failed

**Table S7**. Additive genetic ${\hat{\sigma}^{2}}_{a}$ and residual ${\hat{\sigma}^{2}}_{e}$ variance components for all traits^a^ for both Normandin (a) and Watford (b) study sites using ABLUP and GBLUP^b^. Levels of statistical significance are shown by asterisks^c^.

| Variance components | Recovery | Relative resilience | Resilience | Resistance | Height | DBH | Acoustic velocity | Wood density | EW density | LW density | EW area | LW area |
| --- | --- | --- | --- | --- | --- | --- | --- | --- | --- | --- | --- | --- |
| **a)** | **Normandin** |  |  |  |  |  |  |  |  |  |  |  |
|  | **ABLUP** |  |  |  |  |  |  |  |  |  |  |  |
| ${\hat{\sigma}^{2}}_{e}$ | 0.03 (0.01) | 0.05 (0.01) | 0.08 (0.01) | 0.03 (0.00) | 8512.04 (1881.36) | 355.58 (77.69) | 0.07 (0.02) | 476.73 (105.70) | 331.77 (81.76) | 1617.22 (210.51) | 9680931.27 (1903430.28) | 222853.79 (41134.95) |
| ${\hat{\sigma}^{2}}_{a}$ | 0.01*** (0.01) | 0.02*** (0.01) | 0.03** (0.01) | 0.01* (0.00) | 6713.41*** (2555.41) | 279.52*** (105.29) | 0.06*** (0.02) | 375.43*** (143.56) | 335.02*** (116.37) | 232.60 (190.49) | 5783193.23*** (2438986.61) | 123524.92*** (51434.74) |
|  | **GBLUP** |  |  |  |  |  |  |  |  |  |  |  |
| ${\hat{\sigma}^{2}}_{e}$ | 0.04 (0.01) | 0.05 (0.01) | 0.09 (0.01) | 0.03 (0.00) | 8127.33 (1636.81) | 348.28 (68.83) | 0.08 (0.01) | 485.19 (90.26) | 364.71 (67.95) | 1552.96 (203.17) | 9655583.54 (1721627.12) | 193550.65 (40136.40) |
| ${\hat{\sigma}^{2}}_{a}$ | 0.01*** (0.01) | 0.01*** (0.01) | 0.02** (0.01) | 0.01* (0.00) | 7174.13*** (2329.43) | 288.21*** (96.45) | 0.05*** (0.02) | 354.32*** (122.17) | 287.25*** (93.34) | 295.10* (190.92) | 5768337.67*** (2226266.63) | 161789.32*** (56785.95) |
| **b)** | **Watford** |  |  |  |  |  |  |  |  |  |  |  |
|  | **ABLUP** |  |  |  |  |  |  |  |  |  |  |  |
| ${\hat{\sigma}^{2}}_{e}$ |  |  |  | 0.03 (0.00) | 7872.87 (1701.28) | 323.89 (55.75) | 0.04 (0.01) | 435.32 (104.38) | 302.17 (71.69) | 1322.59 (235.22) | 13863487.27 (2147159.76) | 243752.82 (40306.42) |
| ${\hat{\sigma}^{2}}_{a}$ |  |  |  | 0.01** (0.00) | 6534.35*** (2303.90) | 144.47** (65.75) | 0.08*** (0.02) | 406.84*** (145.46) | 271.52*** (99.22) | 640.60*** (283.32) | 4668711.38** (2346410.77) | 107708.98*** (46887.61) |
|  | **GBLUP** |  |  |  |  |  |  |  |  |  |  |  |
| ${\hat{\sigma}^{2}}_{e}$ |  |  |  | 0.03 (0.00) | 7733.46 (1467.74) | 316.96 (50.91) | 0.05 (0.01) | 484.45 (86.47) | 333.61 (59.90) | 1159.72 (219.15) | 13368006.60 (2018959.22) | 233265.78 (37035.00) |
| ${\hat{\sigma}^{2}}_{a}$ |  |  |  | 0.01** (0.00) | 6551.21*** (2034.08) | 148.58*** (60.49) | 0.07*** (0.02) | 338.69*** (113.97) | 227.72*** (78.69) | 828.55*** (293.62) | 5095435.18*** (2260410.31) | 116663.78*** (44330.05) |

^a^ See Table 1 for a full description of traits.
^b^ The model fitted for each trait is described in Equation 1.
^c^ Levels of statistical significance: * *P* < 0.05 | ** *P* < 0.01 | *** *P* < 0.001 | † convergence failed.

| Variance components | Recovery | Relative resilience | Resilience | Resistance | Height | DBH | Acoustic velocity | Wood density | EW density | LW density | EW area | LW area |
| --- | --- | --- | --- | --- | --- | --- | --- | --- | --- | --- | --- | --- |
| **a)** | **Normandin** |  |  |  |  |  |  |  |  |  |  |  |
|  | **ABLUP** |  |  |  |  |  |  |  |  |  |  |  |
| Block 1 | 0.00  (-) | 0.00  (-) | 0.00  (-) | 0.00  (-) | 0.00  (-) | 0.00  (-) | 0.00  (-) | 0.00  (-) | 0.00  (-) | 0.00  (-) | 0.00  (-) | 0.00  (-) |
| Block 2 | -0.11  (0.03) | -78.03 (17.86) | -1.25  (3.65) | -0.11  (0.03) | -78.03 (17.86) | -1.25  (3.65) | -0.03  (0.05) | 8.48  (4.23) | 7.29  (3.67) | 12.18  (6.71) | -606.63 (579.62) | -72.47 (87.15) |
| Block 3 | -0.02  (0.03) | -237.41 (18.90) | -4.08  (3.86) | -0.02  (0.03) | -237.41 (18.90) | -4.08  (3.86) | -0.36  (0.06) | 5.51  (4.47) | 5.50  (3.89) | 11.15  (7.06) | -478.32 (612.81) | -107.64 (92.11) |
| Block 4 | -0.18  (0.03) | -236.91 (18.87) | -6.93  (3.85) | -0.18  (0.03) | -236.91 (18.87) | -6.93  (3.85) | -0.37  (0.06) | 13.36  (4.46) | 14.29  (3.88) | 14.25  (7.11) | -1589.20 (612.80) | -302.88 (92.15) |
|  | **GBLUP** |  |  |  |  |  |  |  |  |  |  |  |
| Block 1 | 0.00  (-) | 0.00  (-) | 0.00  (-) | 0.00  (-) | 0.00  (-) | 0.00  (-) | 0.00  (-) | 0.00  (-) | 0.00  (-) | 0.00  (-) | 0.00  (-) | 0.00  (-) |
| Block 2 | -0.11  (0.03) | -82.27 (17.50) | -2.08  (3.59) | -0.11  (0.03) | -82.27 (17.50) | -2.08  (3.59) | -0.03  (0.05) | 8.35  (4.16) | 7.16  (3.65) | 12.38  (6.65) | -685.73 (573.25) | -96.38 (84.71) |
| Block 3 | -0.02  (0.03) | -238.60 (18.50) | -4.11  (3.79) | -0.02  (0.03) | -238.60 (18.50) | -4.11  (3.79) | -0.37  (0.06) | 5.63  (4.40) | 5.82  (3.86) | 10.65  (7.00) | -478.13 (605.65) | -108.67 (89.58) |
| Block 4 | -0.18  (0.03) | -238.73 (18.78) | -6.82  (3.85) | -0.18  (0.03) | -238.73 (18.78) | -6.82  (3.85) | -0.37  (0.06) | 13.02  (4.46) | 14.19  (3.91) | 14.27  (7.08) | -1542.89 (613.59) | -313.65 (90.90) |
| **b)** | **Watford** |  |  |  |  |  |  |  |  |  |  |  |
|  | **ABLUP** |  |  |  |  |  |  |  |  |  |  |  |
| Block 1 |  |  |  | 0.00  (-) | 0.00  (-) | 0.00  (-) | 0.00  (-) | 0.00  (-) | 0.00  (-) | 0.00  (-) | 0.00  (-) | 0.00  (-) |
| Block 2 |  |  |  | -0.03  (0.03) | 13.38  (18.48) | 3.47  (3.47) | -0.12  (0.05) | -1.70  (4.43) | 0.01  (3.67) | 5.31  (7.07) | 1232.31 (700.32) | 71.34  (95.12) |
| Block 3 |  |  |  | -0.01  (0.03) | 39.70  (18.27) | 6.48  (3.43) | -0.07  (0.05) | 8.66  (4.38) | 7.90  (3.62) | 18.97  (6.98) | 1359.79 (691.36) | 188.26 (93.93) |
| Block 4 |  |  |  | 0.01  (0.03) | -12.02 (18.09) | 3.90  (3.40) | -0.13  (0.05) | 7.79  (4.33) | 8.30  (3.59) | 5.54  (6.93) | 628.92 (686.49) | 151.34 (93.20) |
|  | **GBLUP** |  |  |  |  |  |  |  |  |  |  |  |
| Block 1 |  |  |  | 0.00  (-) | 0.00  (-) | 0.00  (-) | 0.00  (-) | 0.00  (-) | 0.00  (-) | 0.00  (-) | 0.00  (-) | 0.00  (-) |
| Block 2 |  |  |  | -0.02  (0.03) | 13.62  (18.25) | 3.72  (3.44) | -0.12  (0.05) | -2.05  (4.45) | -0.28  (3.68) | 5.49  (6.90) | 1268.57 (693.59) | 73.70  (93.96) |
| Block 3 |  |  |  | -0.01  (0.03) | 37.21  (18.18) | 6.47  (3.42) | -0.08  (0.05) | 9.20  (4.43) | 8.37  (3.66) | 20.29  (6.87) | 1403.66 (688.34) | 188.53 (93.37) |
| Block 4 |  |  |  | 0.01  (0.03) | -11.65 (17.89) | 3.70  (3.38) | -0.13  (0.05) | 8.00  (4.36) | 8.51  (3.61) | 6.28  (6.77) | 613.25 (680.45) | 139.15 (92.16) |

**Table S8**. Summary of the estimation and standard error, between parentheses, of the fixed effect of the block at both Normandin and Watford study sites.
